# The Effect of Pendent Groups upon Flexibility in Coordination Networks with Square Lattice Topology

**DOI:** 10.1021/acsmaterialslett.3c00565

**Published:** 2023-08-22

**Authors:** Xia Li, Debobroto Sensharma, Kyriaki Koupepidou, Xiang-Jing Kong, Michael J. Zaworotko

**Affiliations:** Department of Chemical Science, Bernal Institute, University of Limerick, Limerick V94 T9PX, Republic of Ireland

## Abstract

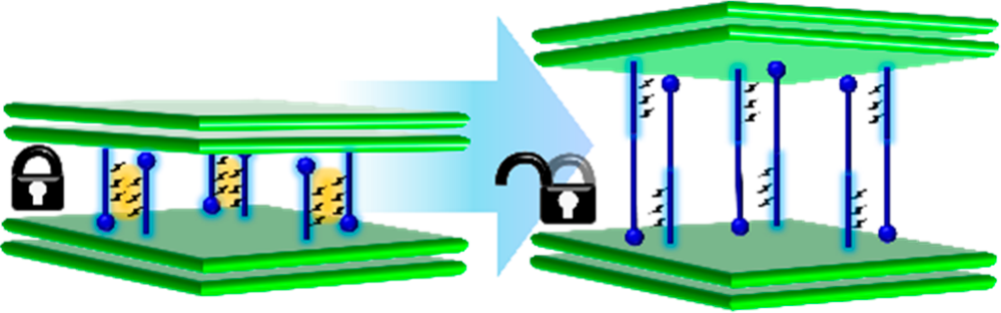

Gas or vapor-induced phase transformations in flexible coordination networks (CNs) offer the potential to exceed the performance of their rigid counterparts for separation and storage applications. However, whereas ligand modification has been used to alter the properties of such stimulus-responsive materials, they remain understudied compared with their rigid counterparts. Here, we report that a family of Zn^2+^ CNs with square lattice (**sql**) topology, differing only through the substituents attached to a linker, exhibit variable flexibility. Structural and CO_2_ sorption studies on the **sql** networks, [Zn(5-**R**ia)(bphy)]_n_, ia = isophthalic acid, bphy = 1,2-bis(pyridin-4-yl)hydrazine, **R** = −CH_3_, −OCH_3_, −C(CH_3_)_3_, -N=N-Ph, and -N=N-Ph(CH_3_)_2_, **2**–**6**, respectively, revealed that the substituent moieties influenced both structural and gas sorption properties. Whereas **2**–**3** exhibited rigidity, **4**, **5**, and **6** exhibited reversible transformation from small pore to large pore phases. Overall, the insight into the profound effect of pendent moieties of linkers upon phase transformations in this family of layered CNs should be transferable to other CN classes.

Flexible coordination networks (CNs), also known as flexible metal–organic frameworks (FMOFs),^1−5^ are an emerging subset of CNs^6,7^ that can undergo stimuli-induced phase transformations. When the external stimulus is gas or vapor pressure, reversible transformation(s) between low- and high-porosity phases can occur, resulting in stepped sorption isotherm profiles with characteristic adsorbate pressure above a threshold or “gate-opening” pressure (*P*_GO_).^8^ Such isotherms can exhibit enhanced working capacities compared with the type I (Langmuir) sorption isotherms typically displayed by rigid materials. Therefore, FMOFs are of potential utility in gas storage^9−12^ or separation^13−16^ and water sorption.^17,18^

Unfortunately, control over the properties of FMOFs is still a challenge. Whereas >118000 entries have been archived in the metal–organic framework (MOF) subset of the Cambridge Structural Database (CSD),^19^ the number of reported FMOFs is much smaller.^3,4^ Nevertheless, that FMOFs can exhibit strong performance for gas/vapor storage or separation makes elucidation of crystal engineering principles for the design of FMOFs a topical area of investigation. The mechanism of flexibility in FMOFs tends to belong to one or more of the following three categories:^3,4^ ligand contortion (e.g., bending, twisting, and/or rotation);^18,20−23^ structural changes in the metal node or molecular building block (MBB)^24^ coordination environment (e.g., deformation, isomerism, or changes in coordination number);^21,23,25−27^ relative motions of individual nets (such as interpenetrated or layered net sliding or expansion).^16,28,29^ There also exist examples of light-modulated adsorbents with photoactive linkers.^30−32^

A strategy to obtain FMOFs involves use of flexible linkers to construct FMOFs.^20,33,34^ In this context, tuning the flexibility of a parent CN through elongation of the backbone or side chain of a linker can enhance flexibility.^35−37^ For example, Kaskel’s group reported the flexible three-dimensional (3D) materials **DUT-49** and **DUT-50**, wherein increasing ligand length led to enhanced flexibility in the resulting frameworks.^38,39^ Alkyl anhydride side-chains on linkers altered the flexibility of **DMOF-1-NH**_**2**_ derivatives, as reported by Cohen’s group.^40^ Webley’s group reported that replacing the rigid BPY (4,4′-bipyridine) linker in Zn(BPDC)(BPY) to the flexible BPP (1,3-bis(4-pyridyl)-propane) analogue in Zn(BPDC)(BPP) through postsynthetic ligand exchange induced flexibility.^41^ Collectively, these reports highlight how pendent groups or ligand extensions can induce flexibility in CNs.^42−45^

CNs that can exhibit subnetwork motion generally exhibit phase transformations involving noncovalent bonds between subnetworks induced via guest inclusion. In the case of two-dimensional (2D) CNs, interlayer expansion can enable adsorption and desorption at gas pressures above and below threshold pressure, respectively. Representative examples include CNs of the elastic layer-structured metal–organic framework (**ELM**) family with square lattice or **sql** topology.^28,33,46−49^ Such structures have relatively weak interactions between the layers, meaning that solvent- or gas-induced expansion can be relatively facile. Such **sql** interlayer expansions are sometimes associated with switching, i.e., transformation from nonporous to porous phases over a narrow pressure range. Void volume changed from 0 to 65.2% in the ELM material **sql-1-Co-NCS**.^16^ However, the reliance of **ELM**s on a specific structural blueprint (metal nodes, counteranions, and linear pyridine ligands) limits their compositional diversity. A strategy to obtain **sql** topology FMOFs with greater diversity involves different linkers; a switching **sql** CN composed of two linkers and Ni(II) cations, **sql-(azpy)(pdia)-Ni**, was recently reported by our group. Regulated by interlayer H-bonds, slippage between adjacent **sql** layers was enabled by the contortion of the pendent ligand (*E*)-5-(phenyldiazenyl)isophthalic acid (H_2_pdia).^18^

Here, we present a new crystal engineering strategy to establish control over expansion between **sql** layers, varying the bulk and length of pendent groups in a family of isophthalic acid linkers. The prototypal structure of this family of networks, [Zn(nipa)(bphy)]_n_, **1**, was previously reported by Chen’s group and is comprised of Zn^2+^ nodes, 5-nitroisophthalic acid (H_2_nipa) and 1,2-bis(pyridin-4-yl)hydrazine (bphy).^50^ That the 5-substituted isophthalate moiety is tunable enables a family of related CNs to be generated by varying the length and steric bulk of the ia substituents. The shorter ia linkers containing methyl, methoxy, and *tert*-butyl groups are available commercially and have been used extensively to generate CNs, with 353, 139, and 212 hits, respectively, archived in the CSD (version 5.43, November 2022).^51^ The bulkier ia substituents studied herein, phenyldiazenyl and (2,4-dimethylphenyl)diazenyl, were obtained by the Wallach reaction,^52^ H_2_pdia and (*E*)-5-((2,4-dimethylphenyl) diazenyl)isophthalic acid (H_2_dpdia) being synthesized in gram scale. Thus far, only 5 and 2 CNs based upon H_2_pdia and H_2_dpdia, respectively, are archived in the CSD. One pdia CN was studied for gas- and vapor-induced flexibility,^18^ whereas two were studied for light-modulated flexibility.^53,54^ Five new **sql** CNs comprised of Zn^2+^, 1,2-bis(pyridin-4-yl)hydrazine (bphy) linkers, and functionalized isophthalic acid linkers: [Zn(mia)(bphy)]_n_ (**2**, mia = 5-methylisophthalate), [Zn(moia)(bphy)]_n_ (**3**, moia = 5-methoxyisophthalate), [Zn(tbia)(bphy)]_n_ (**4**, tbia = 5-(*tert*-butyl)isophthalate), [Zn(pdia)(bphy)]_n_ (**5**, pdia = (*E*)-5-(phenyldiazenyl)isophthalate), and [Zn(dpdia)(bphy)]_n_ (**6**, dpdia = (*E*)-5-((2,4-dimethylphenyl)diazenyl)isophthalate) were prepared, enabling us to study the effect of pendent group substitution upon the flexibility of the CNs.

*Synthesis and structural analysis*. Single crystals of **2**-**6** were prepared by solvothermal reaction of zinc(II) nitrate, H_2_(**R**)ia (5-(**R**)isophthalic acid, **R** = methyl, methoxy, *tert*-butyl, phenyldiazenyl, and 2,4-dimethylphenyldiazenyl, Figure 1a), and azpy ((*E*)-1,2-di(pyridin-4-yl)diazene, Figure 1b) dissolved in *N,N*-dimethylformamide (DMF) and 0.1 M aqueous sodium hydroxide for **2**, **5**, and **6**, or water for **3** and **4**, at 105 °C. Single-crystal X-ray diffraction (SCXRD) studies of the as-synthesized crystals **2**-**6** revealed that **2**, **3**, and **6** had crystallized in *P*2_1_*/n*, **4-α** and **4-α′** in *P*2_1_2_1_2_1_, **4-β** in *P*2_1_, and **5** in *P*2_1_2_1_2 (Figure 1c, Table S1). As previously reported for **1**, azpy underwent *in situ* reduction to bphy during synthesis (Figure 1b).^55^ The mononuclear, tetrahedral molecular building blocks (MBBs)^24^ of **2**-**6** are comprised of Zn^2+^ cations coordinated to two N-donor atoms from two separate bphy ligands and two monodentate carboxylate O-donor atoms from separate isophthalate ligands. The general formula for this family of compounds is [Zn((**R**)ia)(bphy)]_n_ (**R** = methyl, methoxy, *tert*-butyl, phenyldiazenyl, and 2,4-dimethylphenyldiazenyl, Figure 1a,c). **2**-**6** each exhibit layered structures with **sql** topology, the square comprised of bphy ligands and parallel isophthalate moieties that form the edges of a quadrilateral with Zn^2+^ centers at the vertices. Interlayer hydrogen bonds were observed in all five materials between hydrazine hydrogen atoms in bphy from one **sql** layer and the uncoordinated oxygen atom in an isophthalate ligand from an adjacent **sql** layer, lengths ranging from *d*_N···O_ = 2.799(5) Å in **2** to 2.849(12) Å in **4-α** (Table S2). These H-bonding interactions enabled the **sql** layers to form bilayers with pendent groups extending orthogonally in opposite directions. The pendant groups interdigitate with adjacent H-bonded bilayers to form a porous structure (Figure 1d). Under the same synthetic conditions used for **2**-**6**, isophthalic acid (ia) did not yield the same bilayer structure (Figure S1).

**Figure 1:**
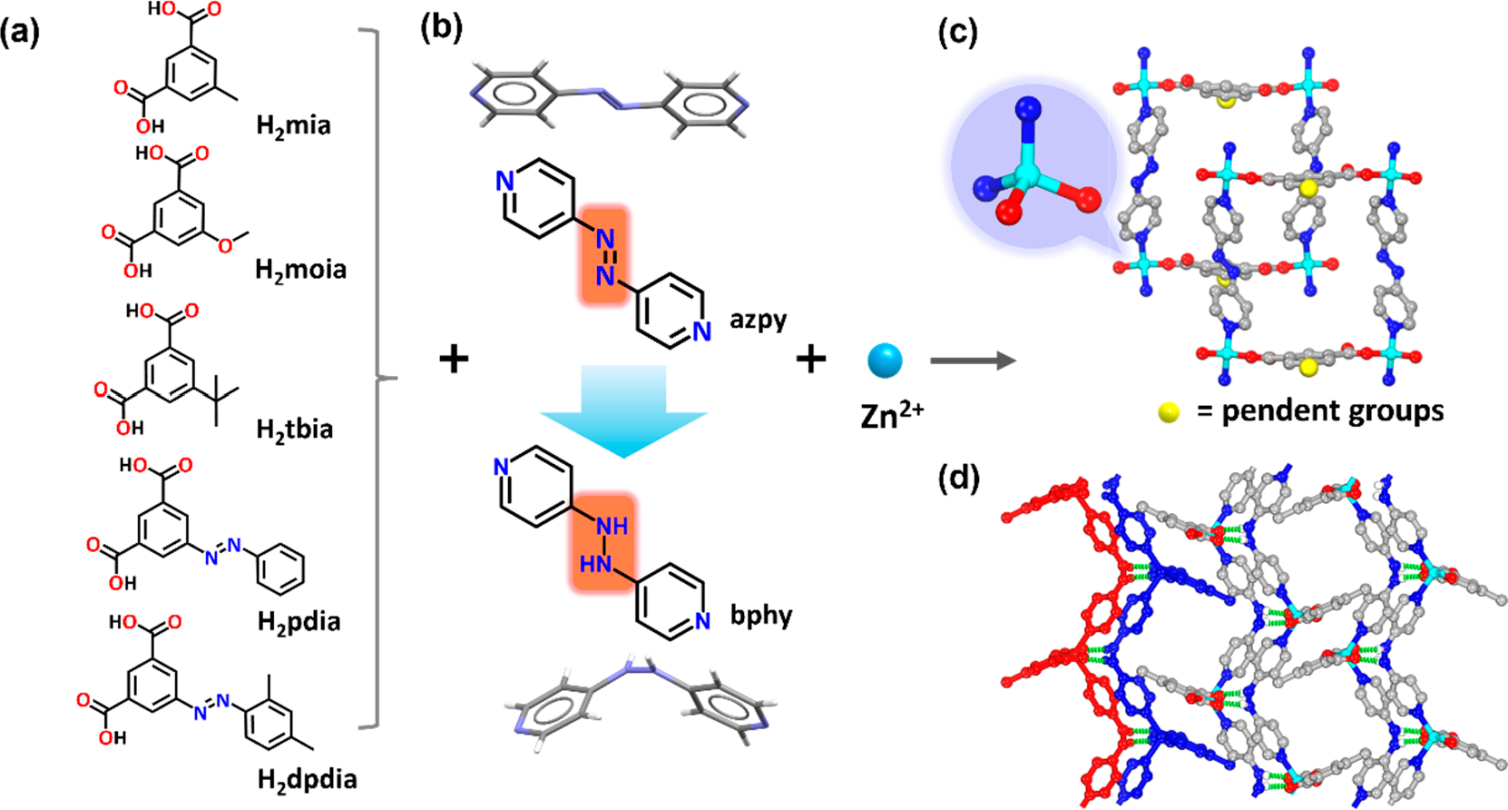
(a) The five functionalized isophthalic acid (ia) ligands studied herein: 5-methylisophthalic acid (H_2_mia); 5-methoxyisophthalic acid (H_2_moia); 5-(*tert*-butyl)isophthalic acid (H_2_tbia); (*E*)-5-(phenyldiazenyl)isophthalic acid (H_2_pdia); and (*E*)-5-((2,4-dimethylphenyl)diazenyl)isophthalic acid (H_2_dpdia). (b) *In situ* reduction of 1,2-di(pyridin-4-yl)diazene (azpy) to 1,2-bis(pyridin-4-yl)hydrazine (bphy) occurred during the synthesis of **sql** topology CNs. (c) The tetrahedral coordination environment of the zinc cations in the CNs studied herein. (d) Interlayer hydrogen bonds (green dashed line) connect two **sql** layers (red, blue) to form bilayers; crystal packing of these bilayers is directed by interdigitation (partial hydrogen atoms omitted for clarity).

The pendent moieties influence the pore dimensions of each compound, with **1**-**6** exhibiting different guest-accessible void volumes in their as-synthesized structures (denoted as -**α**, Figure S2). While retaining the same topology and bilayer architecture, the steric bulk of these five pendent groups resulted in pores of different dimensions. Disordered solvent in the pores were not modeled during structure refinement; a solvent mask^56^ was applied. The guest-accessible void spaces calculated by PLATON^57^ in **1**, **2**, **3**, **4-α**, **5-α**, and **6-α** were 21.6%, 22.5%, 23.9%, 45.4%, 40.5%, and 35.7%, respectively (Figure S2). An incongruence of incremental guest-accessible void space was observed in **4-α**, which was caused by the large volume of the relatively short *tert*-butyl group, which allowed the inclusion of additional guests in the gap between the *tert*-butyl group and the adjacent **sql** plane. In the other five compounds, spaces of this type were not observed between the terminal group and adjacent **sql** layers. The bulk purity of each as-synthesized phase was confirmed by powder X-ray diffraction (PXRD) through comparison with the PXRD pattern calculated from the corresponding SCXRD structure (Figure S3).

*Activation*. Activation was conducted by heating to 333 K under a vacuum after performing solvent exchange using dichloromethane (DCM). **1**, **2**, and **3** did not show any appreciable change in their crystal structures as determined by PXRD (Figure S3). However, **4**-**6** showed marked PXRD pattern changes because of phase transformation to low-porosity “-**β**” phases, **4-β**, **5-β**, and **6-β**, respectively. Notably, upon heating without solvent exchange at 343 K for 30 min, **4-α** transformed to a partially solvated phase with intermediate porosity, **4-α′**, while **5** and **6** did not show corresponding intermediate phases.

The relative bulk of the ia ligands with pendent groups was evaluated by the SCXRD determined distance between the C atom at the 5- position of ia and the terminal O (**1**) or H atom (**2**-**6**) of the pendent moiety. Distances were found to be 2.318(4) Å for nipa^2–^, 2.060(3) Å for mia^2–^, 3.180(2) Å for moia^2–^, 3.354(6) Å for tbia^2–^, 7.228(3) Å for pdia^2–^, and 8.099(5) Å for dpdia^2–^ (Figure S4).

Analysis of the SCXRD structures revealed that **1**-**3** and **4-β** are isomorphous, their unit cell volumes being 2162.87, 2137.5(3), 2227.66(17), and 2270.2(2) Å^3^, respectively (Table S1). That **4-β** is comprised of a bulkier ligand (tbia^2–^) means that it exhibits cavities with voids of 12.4%, while **1**-**3** have pores with voids of 21.6%, 22.5%, and 23.9%, respectively (Figures S2 and S5). Among these four compounds, only **4** was found to exhibit solvent-triggered phase changes involving clay-like **sql** bilayer expansion, resulting in three distinct phases, **4-β**, **4-α′**, and **4-α**. In contrast, the structures of **1**-**3** were unaffected by activation (Table S1).

Further structural analysis revealed that, in **1**-**3**, the edge lengths of the squares comprising the **sql** square lattice Zn-to-Zn distances are 10.185(1) and 11.104(1) Å in **1**, 10.189(1) and 11.199(1) Å in **2**, and 10.211(7) and 11.106(8) Å in **3**, along the isophthalate-bridged edge and the bphy-bridged edge, respectively (Table S3). The torsion angles between the pyridyl carbons (at the 4-position) and hydrazine N atoms (∠C–N–N-C) in bphy are 95.9(3)° for **1**, 99.7(5)° for **2**, and 96.3(5)° for **3**. The dihedral angles in mia^2–^ between each carboxyl plane and the aromatic ring plane of the isophthalate moieties are 9.0(2)° and 18.5(2)° for **1**, 10.6(3)° and 20.4(3)° for **2**, and 6.5(4)° and 16.6(3)° for **3**. The perpendicular distances between two **sql** planes (defined by three adjacent Zn^2+^ centers in the same **sql** layer) are 3.351(1), 3.233(1), and 3.574(1) Å in **1**-**3**, respectively (Figure 2a–c).

**Figure 2:**
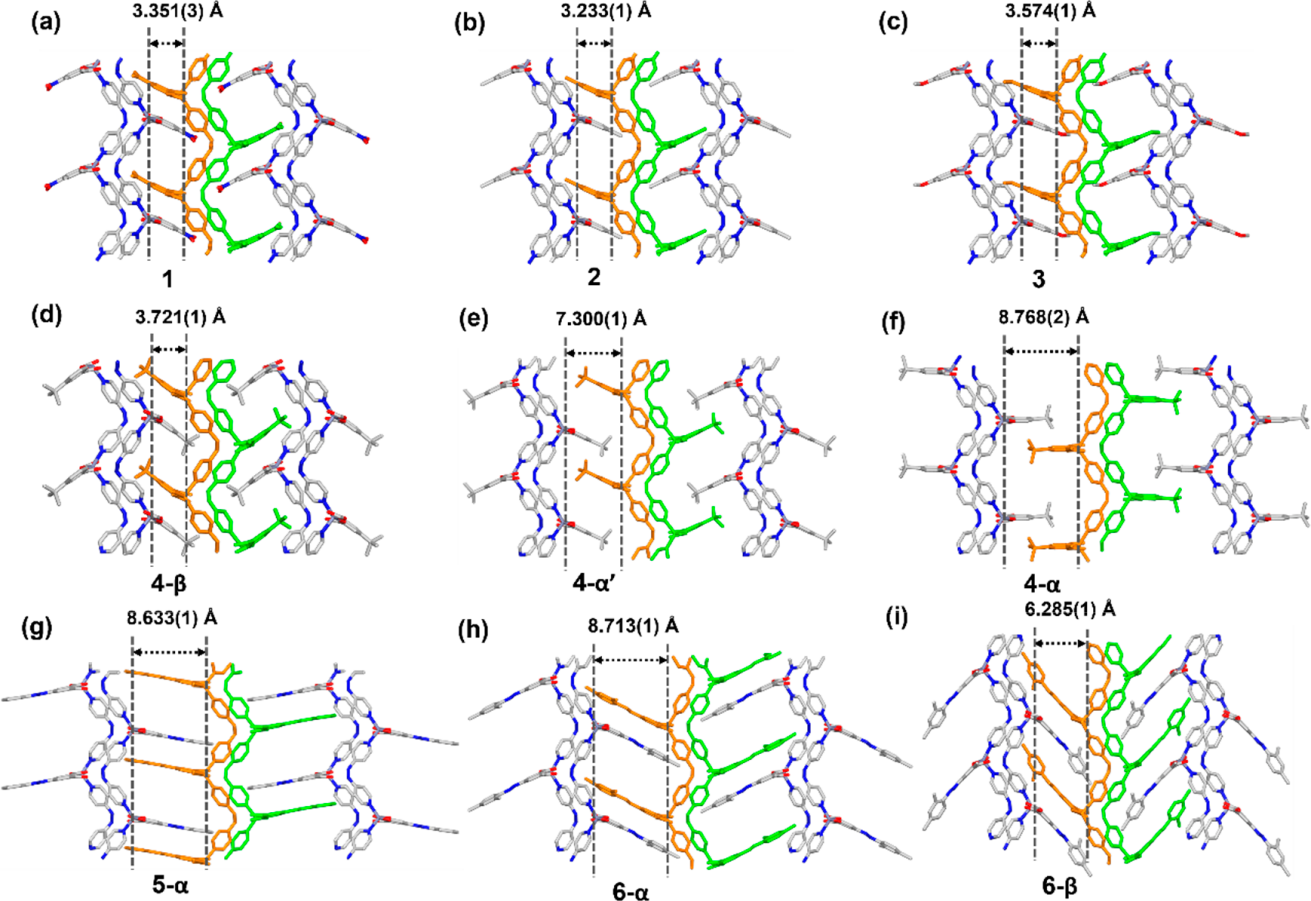
Perpendicular distances between **sql** planes (as defined by three adjacent Zn^2+^ centers in the same **sql** layer) across channels in (a) **1**, (b) **2**, (c) **3**, (d) narrow-pore phase **4-β**, (e) intermediate phase **4-α′**, (f) open phase **4-α**, (g) open phase **5-α**, (h) open phase **6-α**, and (i) narrow-pore phase **6-β** (hydrogen atoms are omitted for clarity).

The interlayer separation for the closed phase of **4**, 3.721(1) Å, changed dramatically to 7.300(1) Å in **4-α′** and 8.768(2) Å in **4-α** (Figure 2d,e). **4-α**, **4-α′**, and **4-β** exhibited cell volumes of 3415.6(5), 3068.09(18), and 2270.2(2) Å^3^ (Table S1). The guest-accessible space was determined to be 45.4% in **4-α** and 36.9% in **4-α′**, while in **4-β** it was only 12.4% (Figure S5). To adapt to the effect of desolvation, bphy and tbia^2–^ displayed flexibility in two dimensions, within and outside of the **sql** plane. Within the **sql** plane, following the phase change from **4-α** to **4-α′** to **4-β**, the edge length between ia-bridged Zn^2+^ cations increased from 10.152(2) to 10.203(1) to 10.252(1) Å, while that between bphy-bridged Zn^2+^ cations decreased from 11.349(2) to 11.209(1) to 11.021(1) Å (Table S3). In each **sql** square lattice, the bphy linker exhibited flexibility with a change in the torsion angle about the hydrazine moiety (∠C–N–N-C) from 101.9(10)° to 98.3(5)° to 89.2(7)°, in **4-α** to **4-α′** to **4-β** (Table S3). Hinge-like deformations of the pendent isophthalate linker were also evident, with the dihedral angle between the carboxyl plane and the isophthalate aromatic ring plane being enlarged from 6.4(8)/10.2(1)° to 9.7(3)/21.2(3)° and from 11.4(5)/27.6(4)° in **4-α** to **4-α′** to **4-β**, respectively (Table S3).

Compared with **1**-**4**, longer pendent moieties are present in **5** and **6** (Figure S4). Crystal fragmentation upon desolvation of **5-α**, as shown in scanning electron microscopy (SEM) images (Figure S6), precluded SCXRD analysis of the activated phase, **5-β**. Nevertheless, PXRD studies on **5** indicated a transformation similar to that observed for **6** (Figure S3), for which SCXRD analysis of the activated phase, **6-β**, was feasible. The SCXRD structures of **6** showed that the pore shape changed from the guest-accessible channels present in **α** to isolated cavities in the **β** phase, as also seen in **4**. The guest-accessible space in **6-α** was determined to be 35.7%, whereas that in **6-β** was 16.4% (Figure S5). However, even though the structural flexibility of **4** was facilitated by the bulky tbia^2–^ moiety, it was a hinge-like motion of pendent moieties dpdia^2–^ in **6** that dominated the expansion between adjacent **sql** bilayers. During the phase transformation from **6-α** to **6-β**, the hinge-like motion is evidenced by a change in the dihedral angles of the carboxyl plane and the isophthalate aromatic ring plane: 8.4(4)/22.8(4)° in **6-α** and 18.1(4)/29.8(4)° in **6-β** (Table S3). Furthermore, additional twisting of the dpdia^2–^ ligand was observed around the azo bond, with the dihedral angle between the isophthalate aromatic ring plane and *m*-xylyl aromatic ring varying from 5.2(3)° to 31.7(4)° in **6-α** and **6-β**, respectively (Table S3). Therefore, the diazo moiety can be an additional source of structural flexibility in **6** compared to **4**. Triggered by solvent molecules, this linker deformation of dpdia^2–^ in **6** resulted in a decrease of the perpendicular distances between two **sql** planes (defined by three adjacent Zn^2+^ centers in the same **sql** layer), with distances of 8.713(1) Å in **6-α** and 6.285(1) Å in **6-β** (Figure 2h,i). **1**-**6** can therefore be classified into two groups by the effect of the various side chains incorporated into each structure. The materials with bulkier (**4**) or longer (**5** and **6**) side chains exhibited flexible behavior, with **5** and **6** containing an additional flexible element, the diazo moiety, which can induce hinge-like motion and further facilitate **sql** bilayer expansion. **1**-**3** did not exhibit layer contraction.

*Analysis of flexibility*. Further analysis of the as-synthesized phases indicated that noncovalent interactions between isophthalate moieties in neighboring **sql** layers play a role in regulating the flexibility of each framework (Figure 3a–d). In the isomorphous structures **1** & **2** and **3** & **4-β**, π···π, C–H···π, and C–H···O interactions exist (Table S4). Specifically, a π···π interaction in each phase was exhibited by a pair of offset-stacked isophthalate rings from adjacent **sql** layers, with *d*_Centroid···Centroid_ = 4.137(4) Å in **1**, 4.176(1) Å in **2**, 3.811(1) Å in **3**, and 4.256(2) Å in **4-β**. C–H···π interactions from the terminal C atom of isophthalate linkers and a pyridyl ring of bphy linkers were found in **2**, **3**, and **4-β**, with average distances of *d*_C···Centroid_ = 3.543, 3.564, and 3.745 Å, respectively. Additionally, C–H···O interactions were also observed in all four compounds. In **1**, **2**, **3**, and **4-β**, four, two, three, and four pairs of C–H···O interactions were found with average distances *d*_C···O_ of 3.195, 3.283, 3.448, and 3.740 Å, respectively. When comparing the distances of the three types of noncovalent interactions measured in **1**-**3** vs **4-β**, **4-β** was found to exhibit longer distances, indicative of a relative weakening of noncovalent interactions. These weaker interactions might explain why guest-triggered **sql** interlayer motion was observed in **4** but not in **1**-**3**.

**Figure 3:**
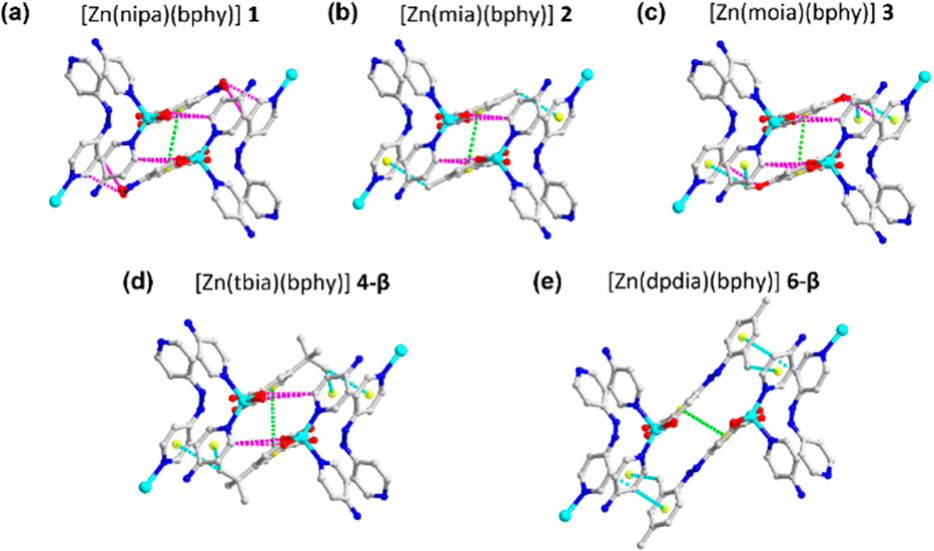
Noncovalent interactions between adjacent sql layers with highlighted π···π in green dashed lines, C–H···π interactions in blue dashed lines, and C–H···O interactions in magenta dashed lines in (a) **1**, (b) **2**, (c) **3**, (c) **4-β**, and (d) **6-β**. (Zn, O, N, C, and created centroid atoms are represented by light blue, red, dark blue, gray, and yellow, respectively. Hydrogen atoms are omitted for clarity.).

The presence of bulkier substituents in **5** and **6** impacts noncovalent interactions between pairs of **sql** layers that define the channels in their respective **β** phases (Table S4). Since similar lengths of longer pendent moieties are found in **5** and **6**, 7.228(3) Å for pdia^2–^ and 8.099(5) Å for dpdia^2–^, respectively (Figure S4), and the PXRD patterns of **5** and **6** are similar, **5** was expected to be amenable to transformation like **6**. In **6-β**, although pairwise stacking between isophthalate moieties in neighboring **sql** layers was observed, the moieties present in the side chains played a more active role in determining the crystal packing. A π···π interaction between a pair of offset stacked isophthalate rings of dpdia^2–^ ligands from adjacent **sql** layers with distances of *d*_Centroid···Centroid_ = 3.825(1) Å and C–H···π interactions between *m*-xylyl moieties and bphy pyridyl rings, with distances of *d*_C···Centroid_ = 4.110(6), 4.214(5), and 4.271(7) Å, were observed (Figure 3e). Notably, most of the average distances associated with π···π, C–H···π, and C–H···O interactions in **4-β** (4.256, 3.745, and 3.740 Å) and **6-β** (3.825, 4.198 Å, C–H···O interaction was not observed) are longer than the corresponding distances in **1** (4.137 and 3.195 Å, C–H···π was not observed), **2** (4.176, 3.543, and 3.283 Å), and **3** (3.811, 3.564, and 3.448 Å). Considering the observation of weaker interactions in **4-β** and **6-β** along with the denser packing of **sql** layers in **1**, **2**, and **3**, we postulate that **4**-**6** are more amenable to structural transformation upon solvent removal than **1**-**3**.

*Thermal and Fourier Transform Infrared (FTIR) analysis*. To gain insight into the phase transformations, variable-temperature PXRD (VT-PXRD) tests under a N_2_ atmosphere were conducted on as-synthesized samples of **1**-**6**. During the temperature ramping process, **1**, **2**, and **3** did not exhibit significant peak shifting, and their respective PXRD patterns calculated from their SCXRD structures matched well with experimental data (Figures 4a and S7). **4** exhibited three distinct PXRD patterns. As shown in Figure 4b, the experimental VT-PXRD patterns at 298, 353, and 413 K match well with the calculated PXRD patterns from the SCXRD-determined structures **4-α**, **4-α′**, and **4-β**, respectively (intensity variations could be caused by preferred orientation or different guests, Figure S8). **5** and **6** each exhibited two PXRD patterns in their VT-PXRD data. The experimental PXRD patterns of the as-synthesized phases at 298 K match well with the calculated PXRD patterns from the respective SCXRD structures of **5-α** and **6-α** (Figures 4c,d and S8). When the temperature was increased to 353 K, a PXRD pattern matching the calculated structure of **6-β** was observed (Figure 4f). A similar change was observed from **5-α** at 373 K; however, in the absence of SCXRD data for **5-β**, its structure cannot be directly compared to **5-α**. Thermogravimetric analysis (TGA) revealed gradual weight losses of 12.5% and 11.3% for **2** and **3** from 100 to 150 °C, corresponding to four and three water molecules, respectively, per formula unit (weight losses calculated from SCXRD data are 14.3% and 10.8%, respectively). TGA conducted on **4**-**6** showed 46%, 24%, and 21% weight loss from 120 to 150 °C, corresponding to three DMF molecules in **4**, two DMF molecules in **5** and **6** per formula, respectively (Figure S9). Weight loss above the respective phase-change threshold temperatures can be attributed to framework decomposition occurring above the temperature range covered in VT-PXRD experiments. The presence of guests in the pores of **1**-**6** was also studied by FTIR spectroscopy. FTIR studies of the as-synthesized forms of **1**-**3** indicated the presence of water (O–H stretching at 3369, 3447, and 3452 cm^–1^). In **4**-**6**, C=O stretching bands from DMF were detected at 1677, 1672, and 1663 cm^–1^ (Figure S10).

**Figure 4:**
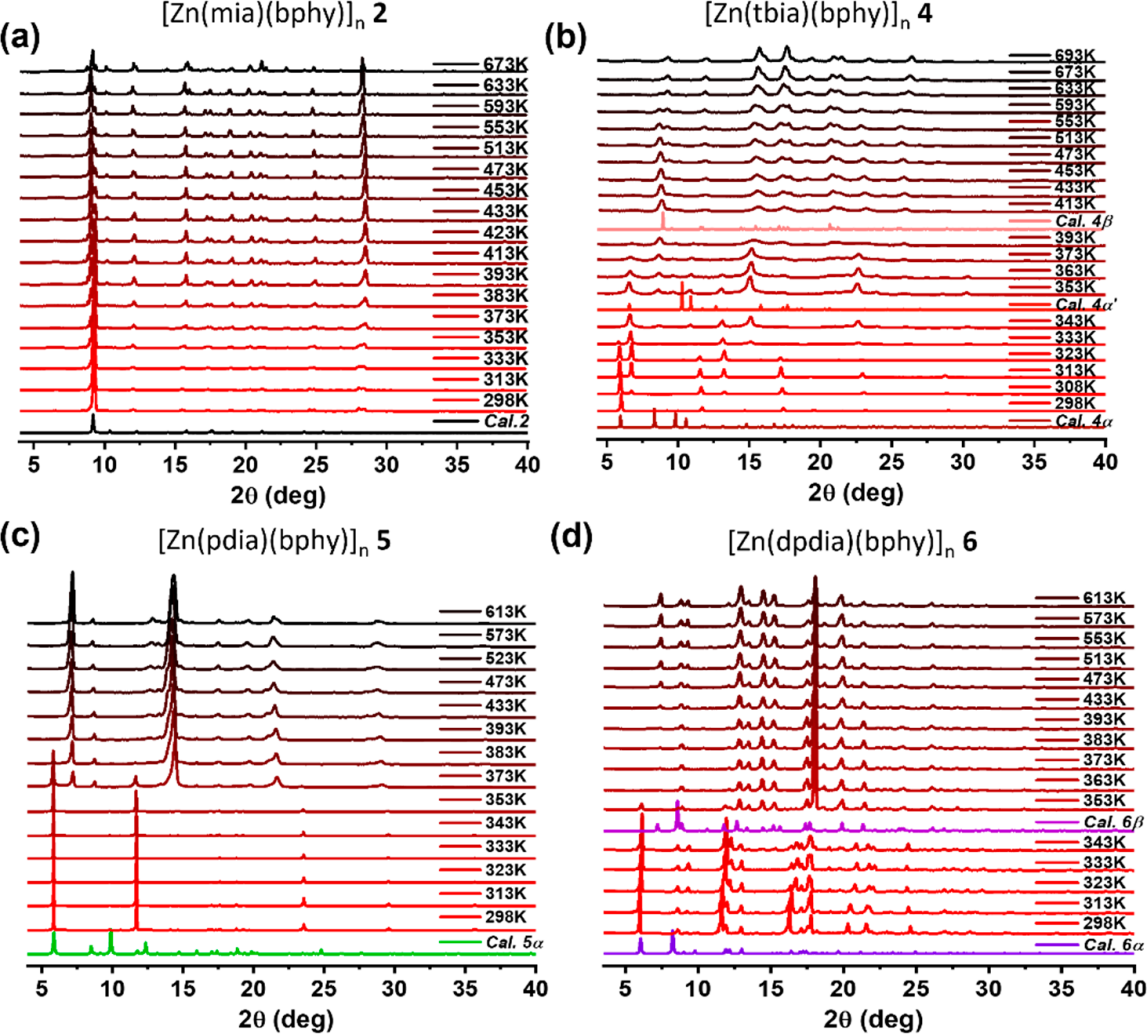
Variable-temperature PXRD patterns of (a) **2** from 298 to 673 K, (b) **4** from 298 to 613 K, (c) **5** from 298 to 613 K, and (d) **6** from 298 to 613 K under N_2_ atmosphere and comparison with calculated PXRD patterns from their SCXRD determined structures.

*Gas sorption*. Since the phase transformations in **4**-**6** can be induced by immersion in liquid solvents, we anticipated that gases might also trigger phase transformation(s). CO_2_ isotherms were collected at 195 K to investigate adsorption characteristics since CO_2_ possesses a small kinetic diameter (3.30 Å) and typically exhibits relatively strong sorbent–sorbate interactions vs N_2_ and Ar. CO_2_ therefore tends to trigger gate opening at 195 K and provide access to the full *P*/*P*_0_ range from 0 to 1.^58^ Sorption experiments conducted on the activated samples of **1**-**6** indicated that **1**-**3** showed type I isotherms characteristic of rigid microporous materials (Figure 5a–c). Conversely, **4**-**6** exhibited stepped isotherms consistent with phase transformations. The CO_2_ uptakes of **1**-**3** at 1 bar and 195 K were determined to be 70, 69, and 62 cm^3^/g with pore sizes of 4.12, 4.16, and 4.11 Å, respectively (Figure S11). In **4** and **6**, “closed-to-open” switching (Type F–IV) isotherms at characteristic gate-opening pressures (*P*_GO_) were observed at *P*_GO_ = 0.04 and 0.12 bar (Figures 5d,f and S12). In **5**, an “open-to-more open” step or Type F–II isotherm was observed at *P*_GO_ = 0.33 bar (Figures 5e and S12). The differences in types of sorption isotherms indicate that a degree of porosity was preserved in the narrow pore phase of **5** after activation, while nonporous phases were obtained in **4** and **6** after activation. The CO_2_ sorption isotherms of **4**-**6** were collected at 273 and 298 K and did not transform to their more open phases (Figure S13). The isosteric heat of adsorption (Qst) of **5** for CO_2_ was calculated from its 273 and 298 K isotherms to be 21.5 kJ/mol (Figure S14). With respect to the desorption branches of the isotherms, the flexible materials **4**, **5**, and **6** exhibited various degrees of hysteresis, while there was no hysteresis for **1**-**3**. The difference in the sorption profiles of **1**-**3** vs **4**-**6** is illustrated by the differences between arbitrary working capacities of CO_2_ at 195 K calculated between 0.1 and 1 bar. The values for **1** (70 cm^3^/g), **2** (69 cm^3^/g), and **3** (62 cm^3^/g) are reduced due to their Type-I uptake characteristics compared to those for **4** (70 cm^3^/g), **5** (174 cm^3^/g), and **6** (154 cm^3^/g), which show flexible isotherms. Grand canonical Monte Carlo simulations of the inclusion of CO_2_ by the porous phases of **4**-**6** suggest no specific binding sites between the CO_2_ molecules and pore walls (Figure S15). Significant N_2_ uptake was found only in **2** (37 cm^3^/g), whereas the other five materials exhibited negligible uptake (Figure S16). The negligible N_2_ uptakes of **1** and **3** can be attributed to their small pore size (Figure S17). For **4**-**6**, there is negligible N_2_ uptake, because transformation to their phases cannot be triggered by N_2_.

**Figure 5:**
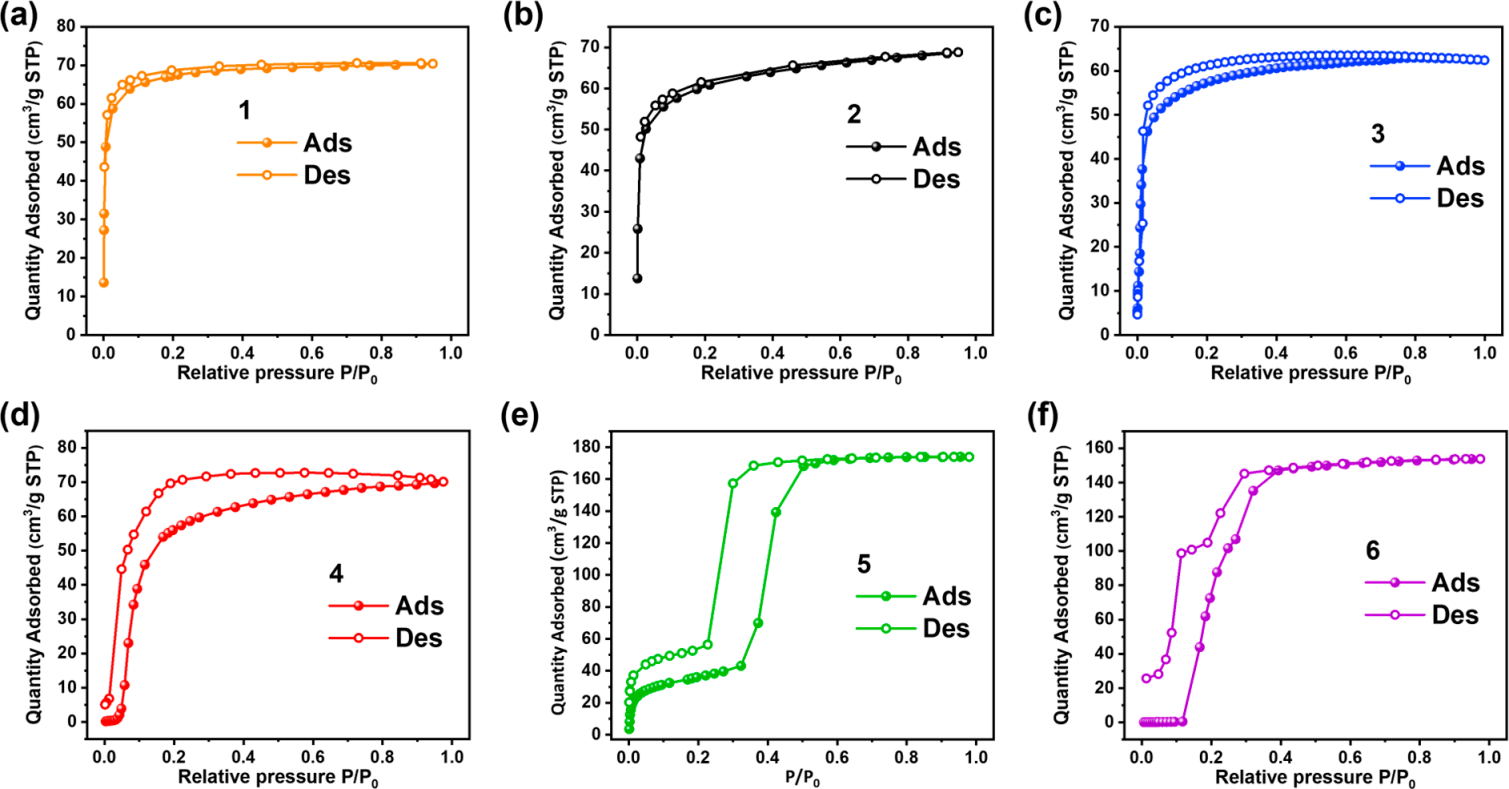
CO_2_ sorption isotherm at 195 K of (a) **1**, orange; (b) **2**, black; (c) **3**, blue; (d) **4**, red; (e) **5**, green; and (f) **6**, purple. Adsorption and desorption branches are indicated by solid and open symbols, respectively.

*Conclusion*. We report a family of six **sql** CNs with interdigitated bilayer packing and different lengths and bulkiness of pendent groups in their ia linkers. Upon desolvation and gas sorption, those CNs with short but bulkier or longer pendent groups exhibited structural transformations between low-porosity phases and high-porosity phases facilitated by both interlayer expansion and linker hinge-like motions. While **sql** layer expansion dominated for **4** (short and bulky pendent moiety), linker hinge-like motion dominated for **5** and **6** (longer pendent groups), as revealed by SCXRD and VT-PXRD experiments. In addition, whereas Type F–II and F–IV CO_2_ sorption isotherms were observed in the flexible **sql** CNs (**4**-**6**), those with smaller pendent groups exhibited rigid structures and Type I CO_2_ sorption isotherms (**1**, **2**, and **3**). That the stimulus-induced properties of **1**-**6** were so profoundly influenced by the systematic substitution of pendent groups offers a platform for exploring the development of sorbents with structural flexibility.

## Supporting Information Available

Materials and methods, supporting figures, supporting tables, and supporting references (PDF)
X-ray data for [Zn(mia)(bphy)]_n_ (**2**) (CIF)
X-ray data for [Zn(moia)(bphy)]_n_ (**3**) (CIF)
X-ray data for [Zn(tbia)(bphy)]_n_ (**4-α**) (CIF)
X-ray data for [Zn(tbia)(bphy)]_n_ (**4-α′**) (CIF)
X-ray data for [Zn(tbia)(bphy)]_n_ (**4-β**) (CIF)
X-ray data for [Zn(pdia)(bphy)]_n_ (**5-α**) (CIF)
X-ray data for [Zn(dpdia)(bphy)]_n_ (**6-α**) (CIF)
X-ray data for [Zn(dpdia)(bphy)]_n_ (**6-β**) (CIF)

## Supplementary Material

tz3c00565_si_001.pdf

tz3c00565_si_002.cif

tz3c00565_si_004.cif

tz3c00565_si_006.cif

tz3c00565_si_007.cif

tz3c00565_si_008.cif

tz3c00565_si_009.cif

tz3c00565_si_010.cif

tz3c00565_si_011.cif
