# Supplementary material for: The Effect of Pendent Groups upon Flexibility in Coordination Networks with Square Lattice Topology
Source: ACS Mater Lett. 2023 Aug 22;5(9):2567–75. doi: 10.1021/acsmaterialslett.3c00565 (PMC10481394; doi:10.1021/acsmaterialslett.3c00565)
Supplement: Supplementary file 1 — tz3c00565_si_001.pdf [file tz3c00565_si_001.pdf]

Supporting Information

The Effect of Pendent Groups upon Flexibility in Coordination Networks with Square Lattice Topology

Xia Li, Debobroto Sensharma, Kyriaki Koupepidou, Xiang-Jing Kong, Michael J. Zaworotko*

Department of Chemical Science, Bernal Institute, University of Limerick, Limerick, V94 T9PX, Republic of Ireland

Context

1. Materials and Materials and Synthesis 2
2. Single-crystal X-ray diffraction (SCXRD) measurements....................4
3. Thermogravimetric analysis (TGA) 4
4. IR spectra Fourier Transform Infrared (FTIR) Spectroscopy 4
5. Powder X-ray diffraction (PXRD) measurements 4
6. Variable Temperature Powder X-ray Diffraction (VT-PXRD) 5
7. Scanning Electron Microscopy (SEM)....................5
8. Gas adsorption measurements. 5
9. Modelling. 6
10. Supporting Figures and Tables 7
11. Reference 31

1. Materials and Materials and Synthesis

All chemicals 5-methylisophthalic acid, 5-methoxyisophthalic acid, 5-(*tert*-butyl)isophthalic acid, and $Zn(NO_3)_2 \cdot 6H_2O$ were obtained commercially and used as received without further purification. Synthesis of ligands (*E*)-5-(phenyldiazenyl)isophthalic acid, (*E*)-5-((2,4-dimethylphenyl)diazenyl) isophthalic acid, (*E*)-1,2-di(pyridin-4-yl)diazene was accomplished by applying previously reported procedures.[1–3]

$[Zn(nipa)(bphy) \cdot 3H_2O]_n$ (**1**):
1 was synthesized by a modification of previously reported procedures.[4] A mixture of $Zn(NO_3)_2 \cdot 6H_2O$ (0.05 mmol, 14.9 mg), H_2nipa (0.05 mmol, 9.0 mg), and (*E*)-1,2-di(pyridin-4-yl)diazene (azpy) (0.05 mmol, 9.2 mg) in 2 mL of *N, N*-dimethylformamide (DMF) and 1 mL water was added to a 10mL glass vial and heated to 105 °C for 3 days. After the vial was cooled to room temperature, the mother liquid was decanted, and the resulting as-synthesized crystals were harvested without solvent exchange. Elemental analysis result: C, 41.18%; N, 13.96%; H, 3.56%. Calculated composition: C, 41.97%; N, 13.60%; H, 3.69%. Then the crystals were exchanged with dichloromethane (DCM) for 2 days and heated under vacuum at 60 °C for 10h for activating samples.

$[Zn(mia)(bphy) \cdot 4H_2O]_n$ (**2**):
Synthesis: A mixture of $Zn(NO_3)_2 \cdot 6H_2O$ (0.05 mmol, 14.9 mg), H_2mia (0.05 mmol, 9.0 mg), and (*E*)-1,2-di(pyridin-4-yl)diazene (azpy) (0.05 mmol, 9.2 mg) in 2 mL of *N, N*-dimethylformamide (DMF) and 2 drops of sodium hydroxide water solution (0.1 M) was added to a 10mL glass vial and heated to 105 °C for 3 days. After the vial was cooled to room temperature, the mother liquid was decanted, and the resulting as-synthesized crystals were harvested without solvent exchange. Elemental analysis result: C, 45.36%; N, 12.44%; H, 4.30%. Calculated composition: C, 45.44%; N, 11.16%; H, 4.78%. Then the crystals were exchanged with dichloromethane (DCM) for 2 days and heated under vacuum at 60 °C for 10h for activating samples (Yield, ~ 76% based on Zn).

$[Zn(moia)(bphy) \cdot 3H_2O]_n$ (**3**):
Synthesis: A mixture of $Zn(NO_3)_2 \cdot 6H_2O$ (0.05 mmol, 14.9 mg), H_2moia (0.05 mmol, 9.8 mg), and (*E*)-1,2-di(pyridin-4-yl)diazene (azpy) (0.05 mmol, 9.2 mg) in 2 mL of DMF and 1 mL of water was added to a 10mL glass vial and heated to 105 °C for 3 days. After the vial was cooled to room temperature, the mother liquid was decanted, and the resulting as-synthesized crystals were harvested without solvent exchange. Elemental analysis result: C, 45.51%; N, 11.93%; H, 3.81%. Calculated composition: C, 45.62%; N, 11.21%; H, 4.40%. Then the crystals were exchanged with dichloromethane (DCM) for 2 days and heated under vacuum at 60 °C for 10h for activating samples (Yield, ~ 75% based on Zn).

$\{[Zn(tbia)(bphy)] \cdot 3DMF\}_n$ -α (**4-α**):

Synthesis: The synthesis of **4-α** was similar to that of **3** except H_2tbia (0.05 mmol, 11.1 mg) was used instead of H_2moia and 0.05 mL of water was used instead of 1mL. (Yield, ~ 71% based on Zn). Elemental analysis result: C, 52.33%; N, 13.19%; H, 6.79%. Calculated composition: C, 53.83%; N, 14.18%; H, 6.07%.

{[Zn(tbia)(bphy)]·2DMF}$_n$-α' (**4-α'**):
Synthesis: The as synthesized open phase **4-α** was directly heated to 70 °C for 30 minutes to yield **4-α'**.

[Zn(tbia)(bphy)]$_n$ (**4-β**):
Synthesis: The as synthesized open phase **4-α** was exchanged with dichloromethane (DCM) for 2 days and heated under vacuum at 60 °C for 10h to yield **4-β**.

{[Zn(pdia)(bphy)]·2DMF}$_n$-α (**5-α**):
Synthesis: The synthesis of **5-α** was similar to that of **2** except H_2pdia (0.05 mmol, 13.5 mg) was used instead of H_2mia. (Yield, ~ 70% based on Zn). Elemental analysis result: C, 53.90%; N, 16.49%; H, 4.63%. Calculated composition: C, 54.05%; N, 16.82%; H, 4.80%.

[Zn(dpdia)(bphy)]$_n$ (**5-β**):
Synthesis: The as synthesized open phase **5-α** was exchanged with DCM for 2 days and heated under vacuum at 60 °C for 10h to yield **5-β**.

{[Zn(dpdia)(bphy)]·2DMF}$_n$-α (**6-α**):
Synthesis: The synthesis of **6-α** was similar to that of **2** except H_2dpdia (0.05 mmol, 14.9 mg) was used instead of H_2mia. (Yield, ~ 80% based on Zn). Elemental analysis result: C, 55.50%; N, 16.37%; H, 4.92%. Calculated composition: C, 55.41%; N, 16.16%; H, 5.19%.

[Zn(dpdia)(bphy)]$_n$ (**6-β**):
Synthesis: The as synthesized open phase **6-α** was exchanged with DCM for 2 days and heated under vacuum at 60 °C for 10h to yield **6-β**.

2. Single-crystal X-ray diffraction (SCXRD) measurements.

Single-crystal reflection data were collected on a Bruker Quest diffractometer equipped with a Photon 100 detector and IµS microfocus X-ray source (Cu K_{α}, λ = 1.54178 Å; Mo K_{α}, λ = 0.71073 Å). Indexing was performed using APEX3[5] (Difference Vectors method). Absorption correction was performed by the multi-scan method implemented in SADABS.[6] Space group was determined using XPREP implemented in APEX3.[5] Structural solution and refinement against F^2 were carried out using the SHELXT[7] non-linear least squares implemented in Olex2 v1.2.10.[8,9] All non-hydrogen framework atoms were refined with anisotropic parameters, while H atoms were placed in calculated positions and refined using a riding model. Some disordered atoms have been refined isotropically. Disordered water molecules of hydration were located for **2** and **3**. In this case, some of this electron density could be modelled as a water molecule with partial occupancy, however, refinement was unsatisfactory and the atomic displacement parameters were unreasonable. The PLATON SQUEEZE[10] routine was performed to account for the electron density, resulting in satisfactory refinement. In **2**, the electron count/unit cell agreed well with the presence of four water molecules (electron count/cell = 38, 38/10 = 3.8). In **3**, the electron count/unit cell agreed well with the presence of three water molecules (electron count/cell = 29, 29/10 = 2.9). All solvated phases were collected under liquid N_2 flow at temperatures between 100K to 136K to avoid phase transformation caused by guest molecule desorption. After the activation of all compounds, the samples were transferred quickly and coated with oil immediately for avoiding the exposure to atmosphere. Crystallographic data and structural refinement information are listed in Table S1. The structures. both as-synthesized and activated, of **2**, **3**, **6-α** and **6-β** were solved and refined in the $P2_1/n$ space group; **4-α**, and **4-α'** were solved and refined in the $P2_12_12_1$ space group; **4-β** was solved and refined in the $P2_1$ space group, **5-α** was solved and refined in the $P2_12_12$ space group. Crystallographic data for the structures reported in this paper have been deposited with the Cambridge Crystallographic Data Centre as supplementary publication No. CCDC 2243680-2243681, 2243684-2243687, 2243704, 2243706, 2248289- 2248290.

3. Thermogravimetric analysis (TGA)

Thermogravimetric analyses (TGA) were performed under N_2 using a TA Instruments Q50 system. Samples were loaded into aluminium sample pans and heated at 10 K min^{-1} from room temperature to 500 °C.

4. IR spectra Fourier Transform Infrared (FTIR) Spectroscopy

Spectra were obtained by using a FTIR spectrometer (Agilent technologies, Cary 630) in the range of wavelength 4000-650 cm^{-1}.

5. Powder X-ray diffraction (PXRD) measurements

Powder X-ray diffraction patterns were recorded on a PANalytical X'Pert MPD Pro (Cu

Ka, λ = 1.5418 Å) with a 1D X'Celerator strip detector. Experiments were conducted in continuous scanning mode with the goniometer in the theta-theta orientation. Incident beam optics included the Fixed Divergences slit with anti-scatter slit PreFIX module, with a 1/8°divergence slit and a 1/4° anti-scatter slit, as well as a 10 mm fixed incident beam mask and a Soller slit (0.04 rad). Divergent beam optics included a P7.5 anti-scatter slit, a Soller slit (0.04 rad), and a Ni β filter. The data were collected in the range of 2θ = 4 – 40°. Raw data were then evaluated using the X'Pert HighScore Plus™ software V 4.1 (PANalytical, The Netherlands).

6. Variable Temperature Powder X-ray Diffraction (VT-PXRD)

Diffractograms at different temperatures were recorded using a PANalytical X'Pert Pro-MPD diffractometer equipped with a PIXcel3D detector operating in scanning line detector mode with an active length of 4 utilizing 255 channels. The condition of low temperature (-193°C - 450°C) was controlled by Anton Paar LNC Nitrogen Suction Equipment with application of liquid N_2 cooling to the sample chamber. Anton Paar TTK 450 stage coupled with the Anton Paar TCU 110 Temperature Control Unit was used to record the variable temperature diffractograms. The diffractometer is outfitted with an Empyrean Cu LFF (long fine focus) HR (9430 033 7300x) tube operated at 40 kV and 40 mA and CuKα radiation (λ_α = 1.54056 Å) was used for diffraction experiments. Continuous scanning mode with the goniometer in the theta-theta orientation was used to collect the data. Incident beam optics included the Fixed Divergences slit, with a 1/4° divergence slit and a Soller slit (0.04 rad). Divergent beam optics included a P7.5 anti-scatter slit, a Soller slit (0.04 rad), and a Ni-β filter. In a typical experiment, 20 mg of sample was loaded on a zero-background sample holder made for Anton Paar TTK 450 chamber. The data were collected from 4 - 40° (2θ) with a step size of 0.0167113° and a scan time of 50 seconds per step. Crude data were analyzed using the X'Pert HighScore Plus™ software V 4.1 (PANalytical, The Netherlands). The temperatures were controlled from 173K to 298K and 298K to 613 K-693K.

7. Scanning Electron Microscopy (SEM)

Scanning electron microscopy measurements were carried out for the activated samples to investigate particle size. The images were collected on a Hitachi SU-70 instrument, using a 3 kV acceleration voltage and a working distance of 15 mm. Before the measurement, the samples were dispersed on carbon tape attached to SEM stubs and were gold-coated for 50 seconds to enhance surface conductivity.

8. Gas adsorption measurements.

For gas sorption experiments, high-purity gases were used as received from BOC Gases Ireland: N_2 (99.9995%), CO_2 (99.995%) and He (99.9995%). Low-pressure (0-1 bar) CO_2 and N_2 sorption isotherms were measured using Micromeritics 3flex instrument. DCM exchanged samples of $[Zn(nipa)(bphy)]_n$ (**1**), $[Zn(mia)(bphy)]_n$ (**2**),

$[Zn(moia)(bphy)]_n$ (**3**), $[Zn(tbia)(bphy)]_n$ (**4**), $[Zn(pdia)(bphy)]_n$ (**5**), $[Zn(dpdia)(bphy)]_n$ (**6**) were degassed under high vacuum at 60 °C for 10 h on Micromeritics Smart VacPrep instrument. The activated sample (100 mg) was transferred to 3Flex and evacuated at room temperature for 10 hours before the measurements. The temperatures at 77 K and 195 K were maintained using a 4L Dewar filled with liquid nitrogen and a dry ice-acetone mixture respectively.

9. Modelling.

All calculations were carried out using the BIOVIA Materials Studio 2014 (MS) software suite.[11] Sorption of CO_2 in **4**-**6** was simulated using the Sorption module with the Condensed-phase Optimized Molecular Potentials for Atomistic Simulation Studies (COMPASS II)[12] forcefield and the charges automatically assigned. The simulations ran for 1×10^6 equilibration steps and 1×10^7 production steps at 195K. The Adsorption Isotherm task, which makes use of the grand canonical thermodynamic ensemble, was used to simulate the adsorption of the guest molecules at a fixed fugacity; the number of sorbate molecules was varied until equilibrium was reached in a series of fixed pressure runs. Guest molecule adsorption configurations were sampled by the Metropolis Monte Carlo method (MMC),[13] which filters allowable transformations. Trial configurations were generated without bias and transformations that resulted in a state with a higher probability were accepted, while others were rejected. Trial states were governed by the forcefield-derived potential energy. The sites of guest inclusion derived from these calculations are shown in Figure S15.

10.Supporting Figures and Tables

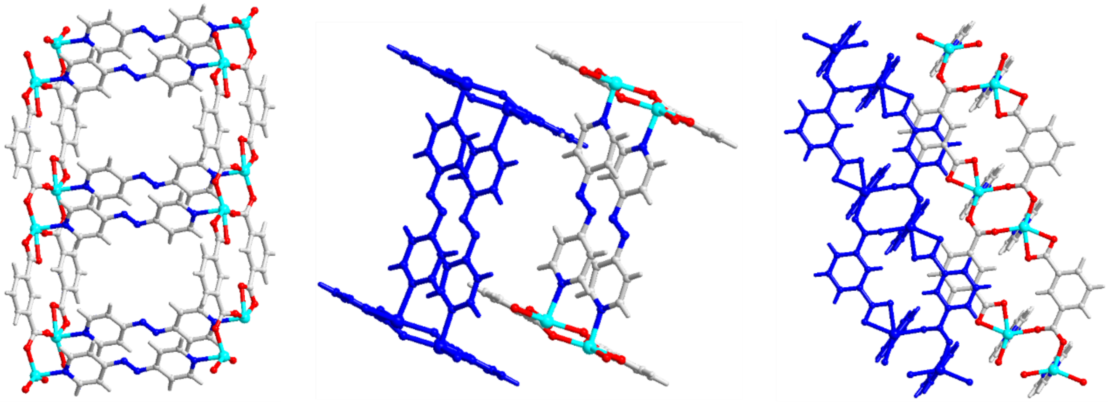

***a* axis** ***b* axis** ***c* axis**

Figure S1. View of [Zn(ipa)(azpy)]$_n$ (ipa = 1,3-isophthalic acid; azpy = 1,2-di(pyridin-4-yl)diazene) along with *a* axis, *b* axis and *c* axis, respectively.

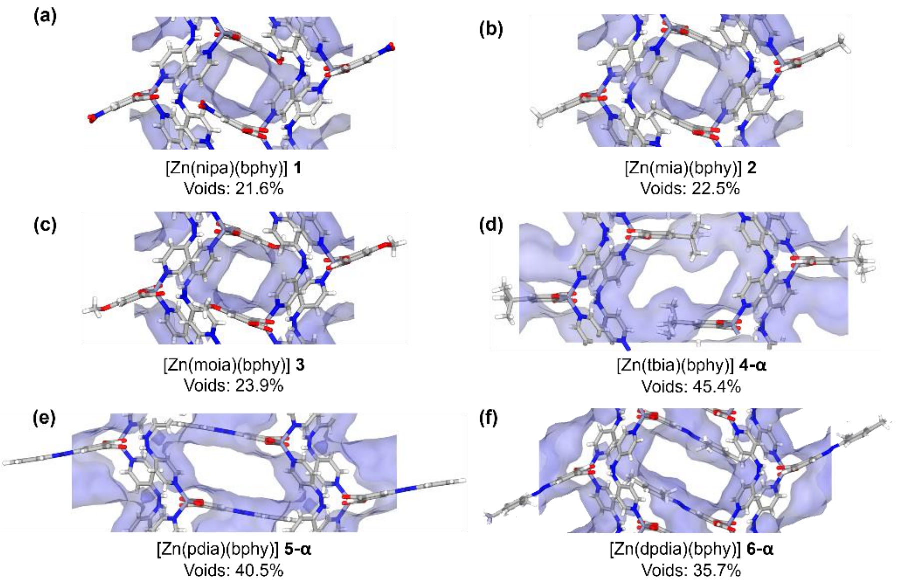

Figure S2. Guest-accessible voids in **1**-**6**: (a) $[Zn(nipa)(bphy)]_n$ (**1**), 21.6%; (b) $[Zn(mia)(bphy)]_n$ (**2**), 22.5%; (c) $[Zn(moia)(bphy)]_n$ (**3**), 23.9%; (d) $[Zn(tbia)(bphy)]_n$ (**4-α**), 45.4%; (e) $[Zn(pdia)(bphy)]_n$ (**5-α**), 40.5%; (f) $[Zn(dpdia)(bphy)]_n$ (**6-α**), 35.7%.

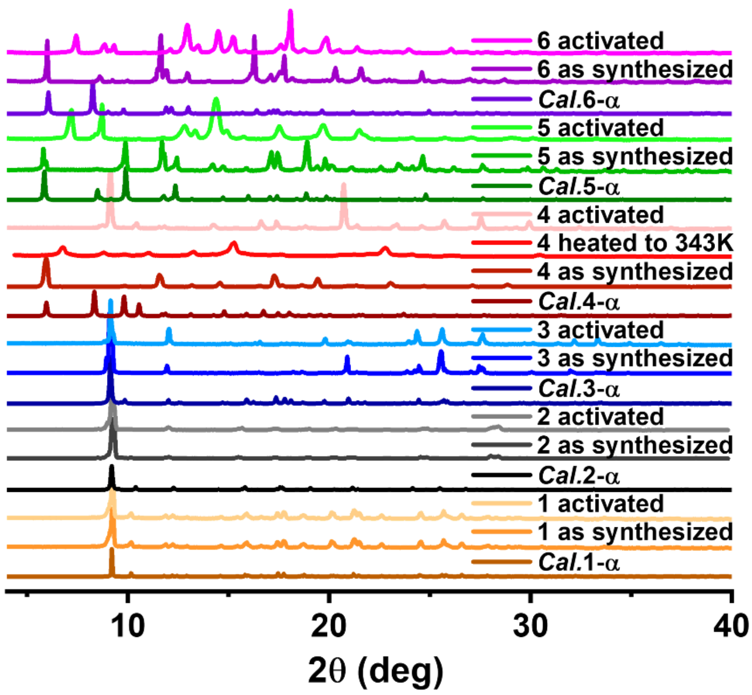

Figure S3. PXRD patterns of as-synthesized and activated samples (Activated samples were obtained by exchanging with DCM for 2 days and then heated under vacuum at 60 °C for 10 h; for **4-α'**, the sample was heated directly to 343K without solvent exchange) in **1** (yellow), **2** (black), **3** (blue), **4** (red), **5** (green), and **6** (purple) and comparison of calculated PXRD patterns from their SCXRD determined structures.

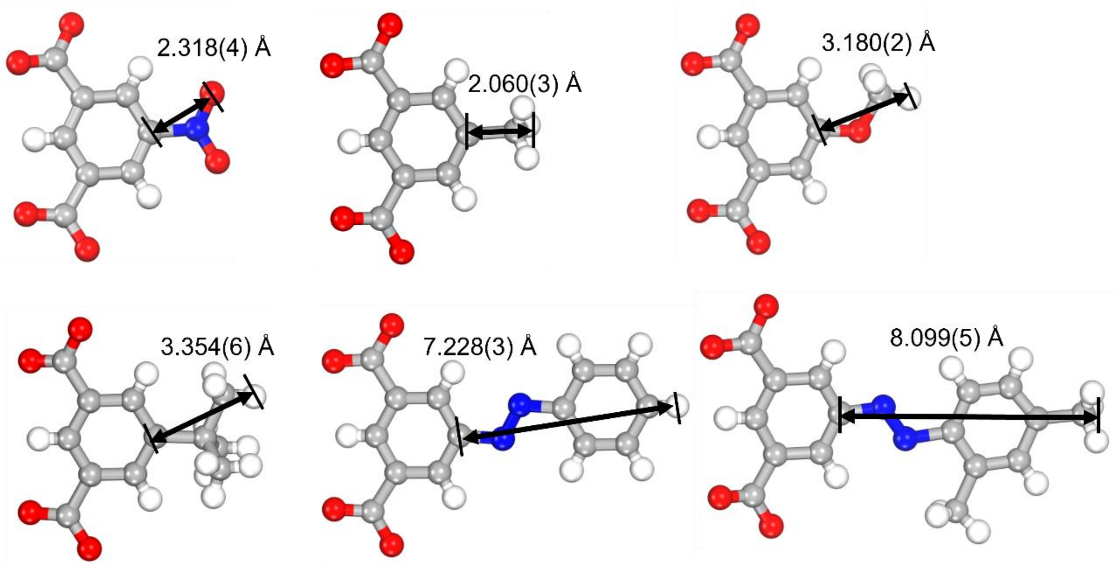

Figure S4. Lengths of pendent groups of six linkers, 5-nitroisophthalic acid (H_2nipa), 5-methylisophthalic acid (H_2mia), 5-methoxyisophthalic acid (H_2moia), 5-(*tert*-butyl)isophthalic acid (H_2tbia), (*E*)-5-(phenyldiazenyl)isophthalic acid (H_2pdia) and (*E*)-5-((2,4-dimethylphenyl)diazenyl) isophthalic acid (H_2dpdia). (Distances were measured between the isophthalate C atom at the 5- position and the terminal H atom in the pendent group.)

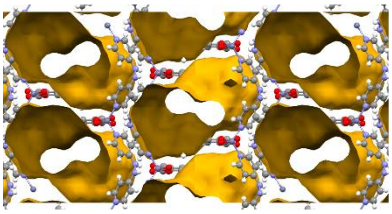

[Zn(tbia)(bphy)] **4-α**
Voids: 45.4%

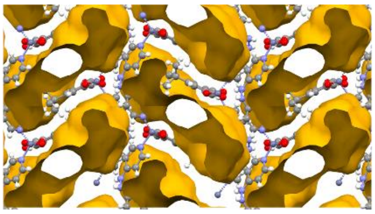

[Zn(tbia)(bphy)] **4-α’**
Voids: 36.9%

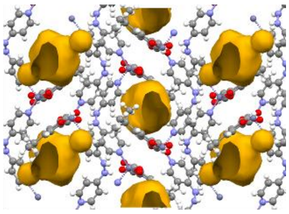

[Zn(tbia)(bphy)] **4-β**
Voids: 12.4%

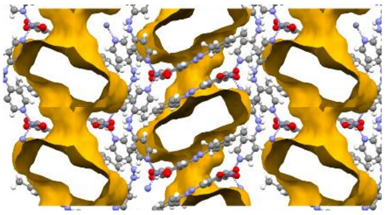

[Zn(dpdia)(bphy)] **6-α**
Voids: 35.7%

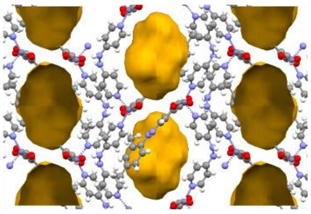

[Zn(dpdia)(bphy)] **6-β**
Voids: 16.4%

Figure S5. Voids of different phases in **4** (**4-α**, 45.4%; **4-α’**, 36.9%; **4-β**, 12.4%); in **6** (**6-α**, 35.7%; **6-β**, 16.4%).

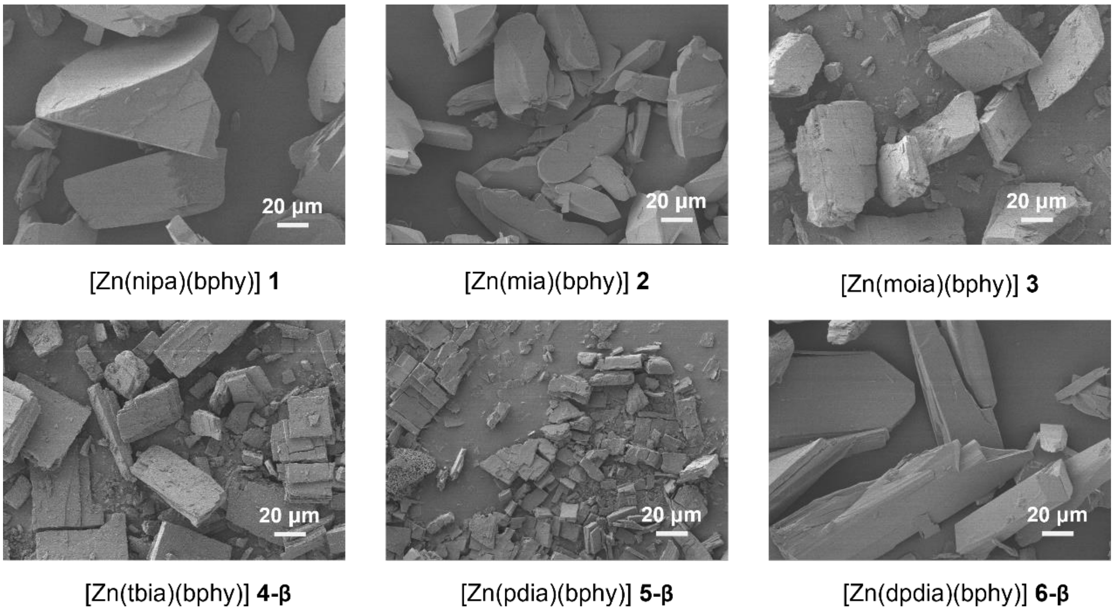

Figure S6. Scanning electron microscopy (SEM) of six activated **sql** networks (**1**, **2**, **3**, **4-β**, **5-β** and **6-β**).

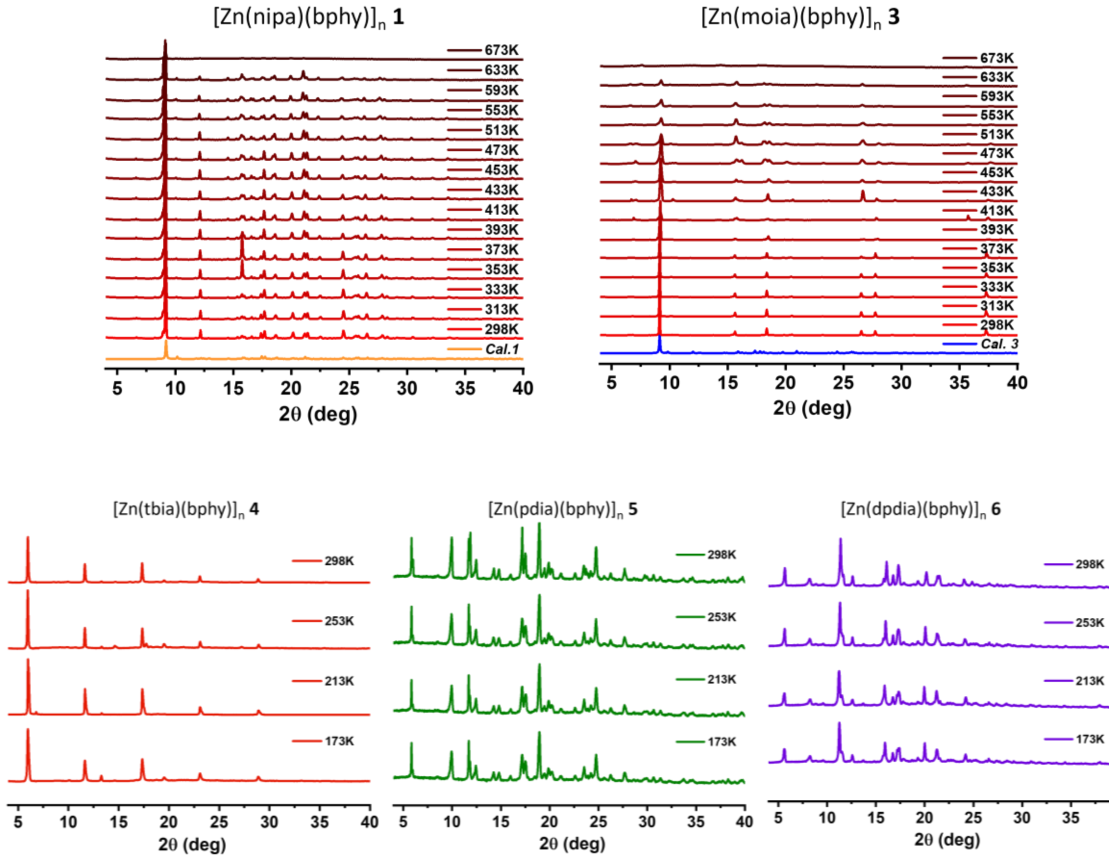

Figure S7. Variable-temperature PXRD (VT-PXRD) patterns of **1** from 298K to 673K and **3** from 298K to 673K under N_2 atmosphere and comparison with calculated PXRD patterns from their SCXRD determined structures. VT-PXRD patterns of **4**-**6** at low temperatures from 173K to 298K under N_2 atmosphere.

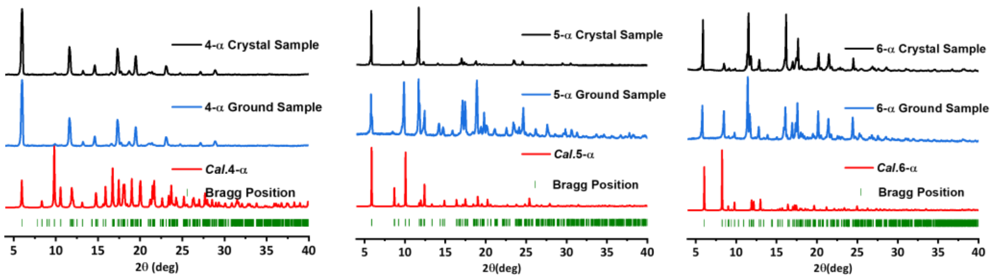

Figure S8. Comparison of the calculated and experimental PXRD patterns of **4-α**, **5-α**, and **6-α** reveals matching of peak positions but variation in the intensities.

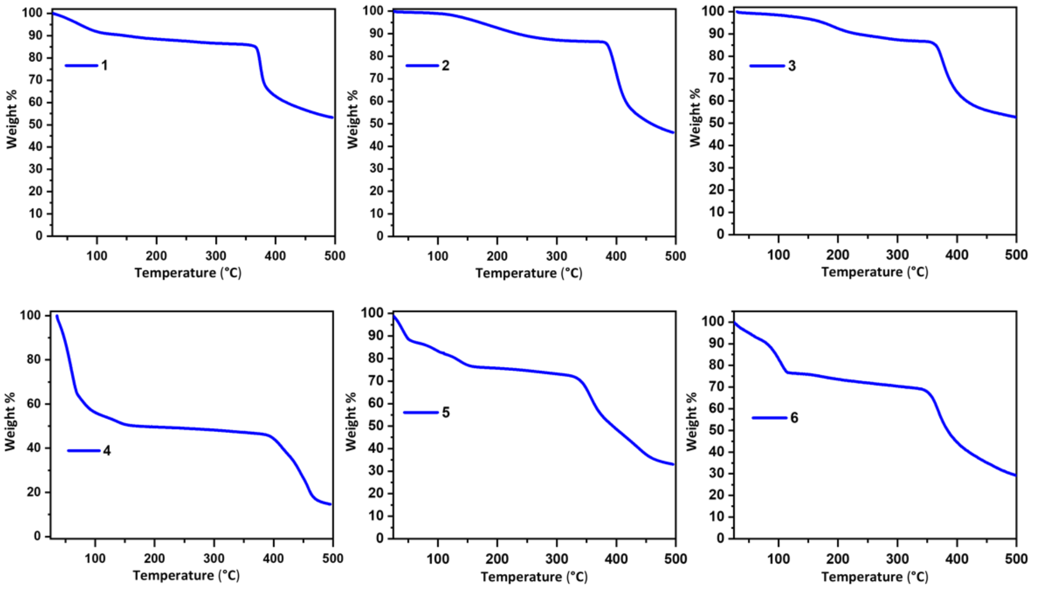

Figure S9. Thermogravimetric analysis was conducted on as-synthesized samples of six **sql** networks: $[Zn(nipa)(bphy)]_n$ (**1**), $[Zn(mia)(bphy)]_n$ (**2**), $[Zn(moia)(bphy)]_n$ (**3**), $[Zn(tbia)(bphy)]_n$ (**4**), $[Zn(pdia)(bphy)]_n$ (**5**), $[Zn(dpdia)(bphy)]_n$ (**6**).

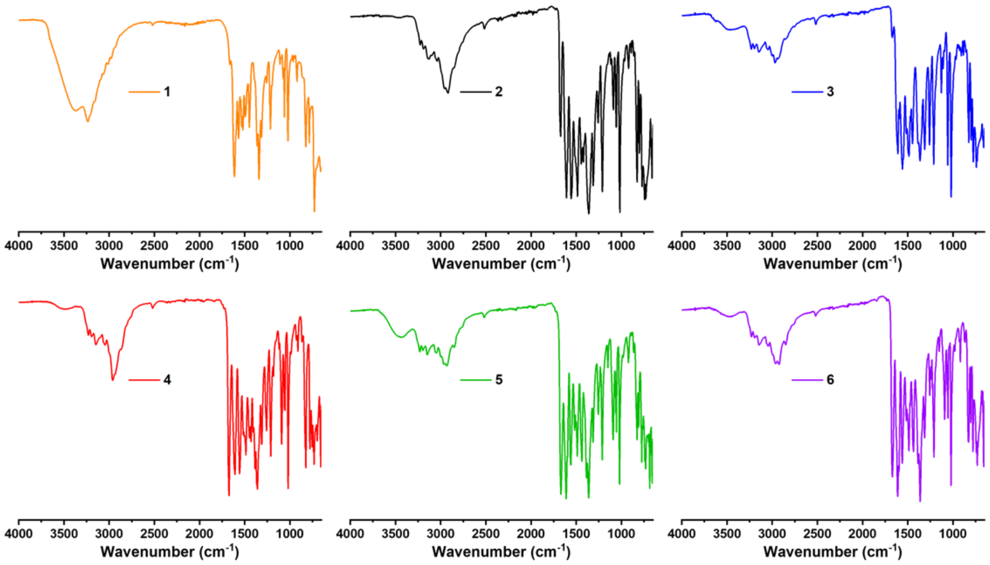

Figure S10. Fourier Transform Infrared (FTIR) spectra of as-synthesized sample:
[Zn(nipa)(bphy)]$_n$ (**1**), IR: ν_{max} (cm^{-1}) = 3369, 1616, 1562, 1342, 1210, 1055, 1022, 818.
[Zn(mia)(bphy)]$_n$ (**2**), IR: ν_{max} (cm^{-1}) = 3447, 2920, 2857, 1671, 1608, 1554, 1480, 1365, 1207, 1014, 726.
[Zn(moia)(bphy)]$_n$ (**3**), IR: ν_{max} (cm^{-1}) = 3452, 1611, 1553, 1359, 1202, 1058, 1021, 740.
{[Zn(tbia)(bphy)]·3DMF}$_n$ (**4**), IR: ν_{max} (cm^{-1}) = 2962, 1677, 1605, 1555, 1365, 1379, 1210, 1089, 1014, 814.
{[Zn(pdia)(bphy)] 2DMF}$_n$ (**5**), IR: ν_{max} (cm^{-1}) = 3161, 2939, 1672, 1613, 1562, 1489, 1362, 1213, 1019, 732.
{[Zn(dpdia)(bphy)] 2DMF}$_n$ (**6**). IR: ν_{max} (cm^{-1}) = 3153, 2962, 2843, 1663, 1608, 1553, 1489, 1362, 1207, 1016, 726.

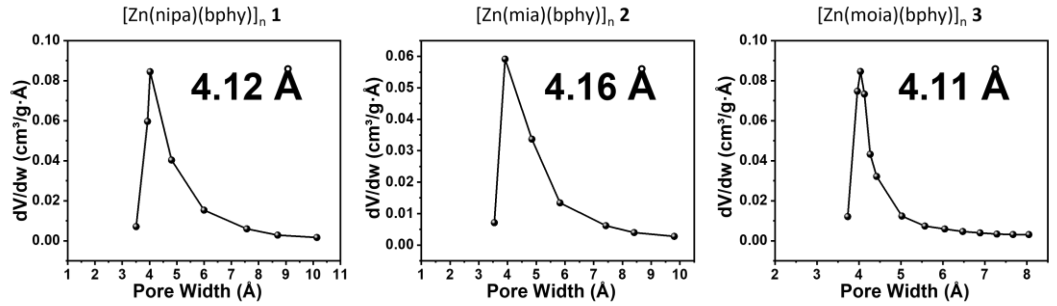

Figure S11. Pore sizes of **1**-**3** that calculated from their 195K CO_2 sorption isotherms.

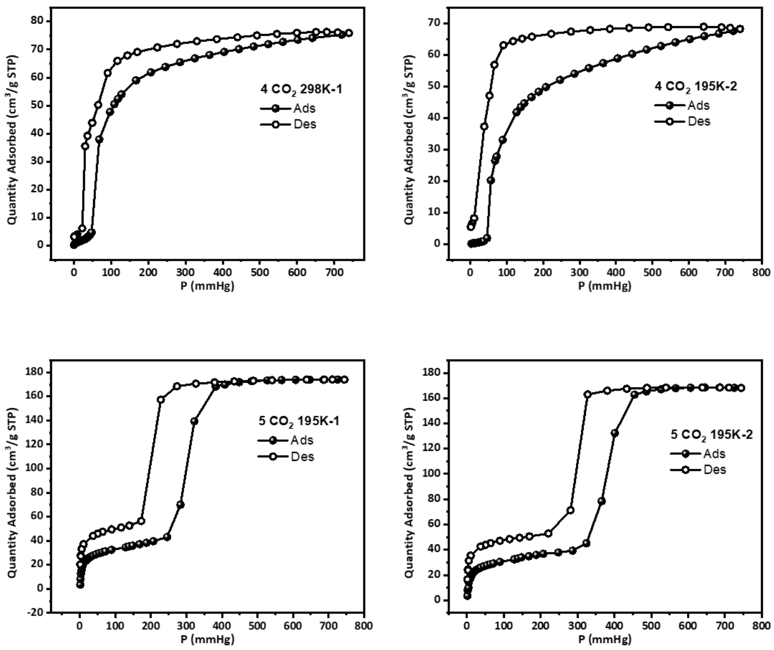

Figure S12. First and second cycles of CO_2 sorption of **4** and **5** at 195K.

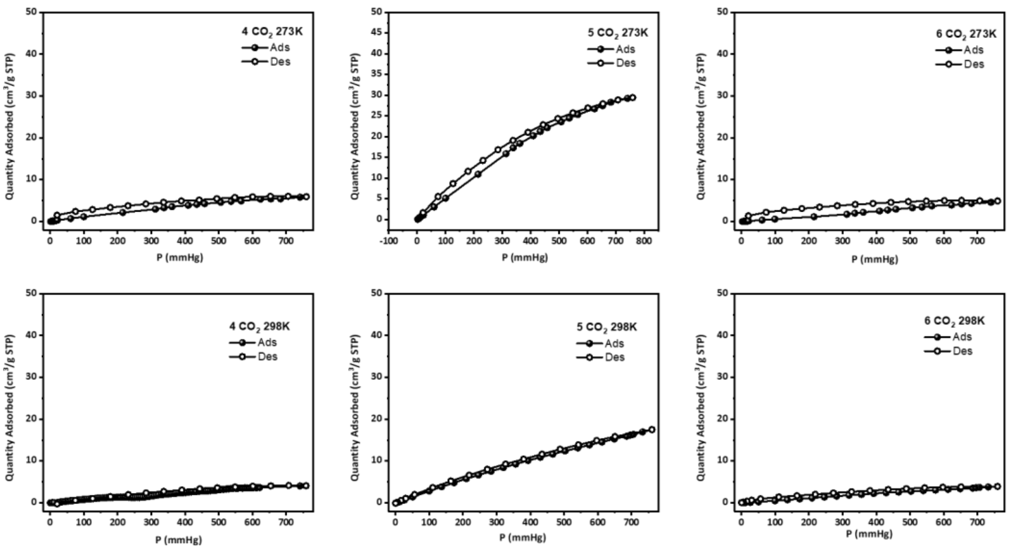

Figure S13. CO_2 sorption isotherms of **4**-**6** at 273K and 298K between 0-1 bar.

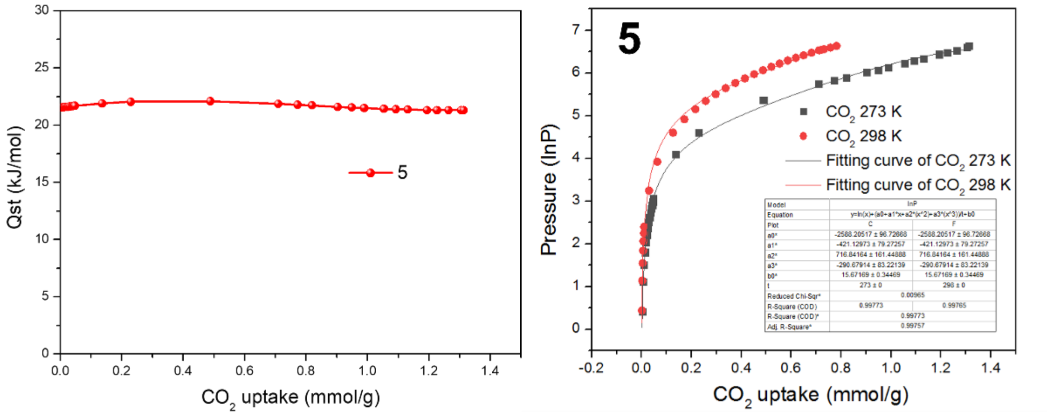

Figure S14. Isosteric enthalpy of adsorption, Qst, of **5** for CO_2 from isotherms collected at 273 K and 298 K was determined to be 21.5 KJ/mol. Realistic values for **4** and **6** could not be obtained because they are non-porous at these temperatures.

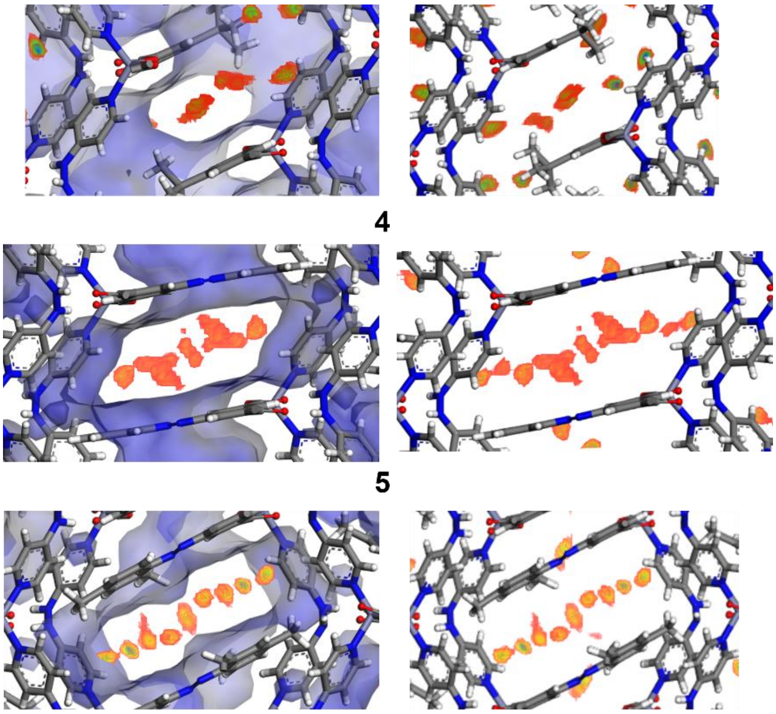

4

5

6

Figure S15. Projection along the channels of porous phases (a) **4**, (b) **5**, (c) **6** showing CO_2 sorption locations (red) in the channel-shaped pore, determined from GCMC calculations at 195K. (Channels were marked with bule (Connolly Surface, solvent radius: 0 Å; Connolly radius: 1.0 Å). Density range of color mapping: 0.1-4.0).

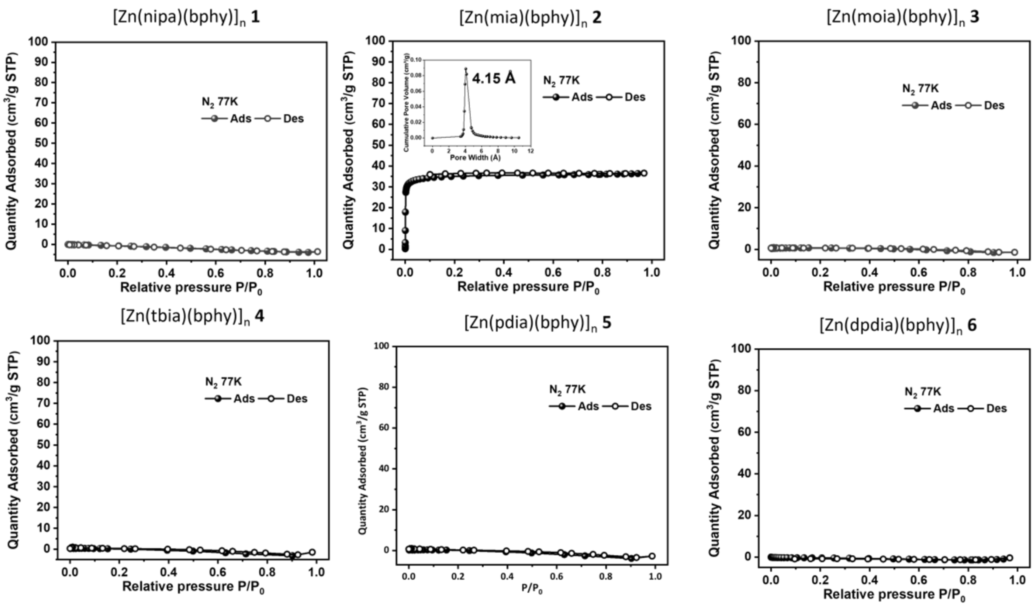

Figure S16. N_2 sorption isotherm at 77K of six activated **sql** networks: [Zn(nipa)(bphy)]$_n$ (**1**), [Zn(mia)(bphy)]$_n$ (**2**), [Zn(moia)(bphy)]$_n$ (**3**), [Zn(tbia)(bphy)]$_n$ (**4**), [Zn(pdia)(bphy)]$_n$ (**5**), [Zn(dpdia)(bphy)]$_n$ (**6**). Pore size of **2** calculated from N_2 isotherm at 77K is 4.15 Å.

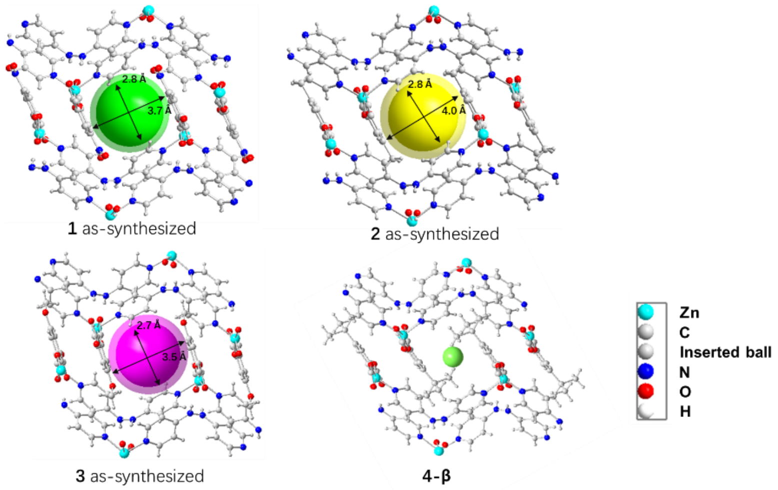

Figure S17. Pore sizes of **1**-**3** and **4-β** measured from single crystal data.

Table S1. Crystallographic data and structure refinement summary for **1**-**6**.

**Compounds**	**2**, as synthesized $[Zn(mia)(bphy)]_n$	**2**, activated $[Zn(mia)(bphy)]_n$	**3**, as synthesized $[Zn(moia)(bphy)]_n$
Identification code	2243680	2248289	2243681
Empirical formula	$C_{19}H_{16}N_4O_4Zn$	$C_{19}H_{16}N_4O_4Zn$	$C_{19}H_{16}N_4O_5Zn$
Formula weight	429.73	429.73	445.73
Temperature/K	100.00	289	136.00
Crystal system	monoclinic	monoclinic	monoclinic
Space group	$P2_1/n$	$P2_1/n$	$P2_1/n$
a/Å	10.1886(9)	10.2102(3)	10.2107(4)
b/Å	11.1985(9)	11.2588(4)	11.1061(5)
c/Å	18.8342(18)	18.7015(7)	19.6495(9)
α/°	90	90	90
β/°	95.913(3)	96.1970(10)	91.3490(10)
γ/°	90	90	90
Volume/Å^3	2137.5(3)	2137.26(13)	2227.66(17)
Z	4	4	4
ρcalcg/cm^3	1.335	1.336	1.329
μ/mm^{-1}	1.178	1.178	1.137
F(000)	880	880	912
Crystal size/mm^3	0.336 × 0.148 × 0.125	0.08 × 0.05 × 0.03	0.192 × 0.183 × 0.165
Radiation	MoKα (λ = 0.71073)	MoKα (λ = 0.71073)	MoKα (λ = 0.71073)
2Θ range for data collection/°	5.686 to 55.044	5.404 to 54.972	5.838 to 54.996
Index ranges	-13 ≤ h ≤ 12, -14 ≤ k ≤ 14, -19 ≤ l ≤ 24	-13 ≤ h ≤ 13, -14 ≤ k ≤ 14, -24 ≤ l ≤ 24	-13 ≤ h ≤ 13, -14 ≤ k ≤ 14, -25 ≤ l ≤ 25
Reflections collected	30091	29814	41503
Independent reflections	4897 [R_{int} = 0.0471, R_{sigma} = 0.0336]	4895 [R_{int} = 0.0407, R_{sigma} = 0.0309]	5082 [R_{int} = 0.0175, R_{sigma} = 0.0100]
Data/restraints/parameters	4952/0/254	4895/0/254	5082/0/263
Goodness-of-fit on F^2	1.107	1.076	1.046
Final R indexes [I>=2σ (I)]	$R1^a$ = 0.0633, $wR2^b$ = 0.1556	$R1^a$ = 0.0359, $wR2^b$ = 0.0803	$R1^a$ = 0.0262, $wR2^b$ = 0.0729
Final R indexes [all data]	$R1^a$ = 0.0689, $wR2^b$ = 0.1580	$R1^a$ = 0.0568, $wR2^b$ = 0.0943	$R1^a$ = 0.0279, $wR2^b$ = 0.0740
Largest diff. peak/hole / e Å^{-3}	1.24/-1.25	0.90/-0.31	1.25/-0.43

$^aR_1=\Sigma||F_o|-|F_c||/\Sigma|F_o|$. $^bwR_2= |\Sigma w(|F_o|^2-|F_c|^2)|/\Sigma|w(Fo^2)^2|^{1/2}$

Table S1. *(Continued)*

**Compounds**	**3**, activated $[Zn(mia)(bphy)]_n$	**4-α**, $\{[Zn(tbia)(bphy)]\cdot 3DMF\}_n$ -α	**4-α'**, $\{[Zn(tbia)(bphy)]\cdot 2DMF\}_n$ -α'
Identification code	2248290	2243704	2243684
Empirical formula	$C_{19}H_{16}N_4O_5Zn$	$C_{31}H_{43}N_7O_7Zn$	$C_{28}H_{36}N_6O_6Zn$
Formula weight	445.73	691.09	618.00
Temperature/K	286	100.00	136.15
Crystal system	monoclinic	orthorhombic	orthorhombic
Space group	$P2_1/n$	$P2_12_12_1$	$P2_12_12_1$
a/Å	10.1777(4)	29.644(3)	10.2033(3)
b/Å	11.2566(5)	10.1524(8)	11.2086(4)
c/Å	18.8808(8)	11.3491(10)	26.8272(10)
α/°	90	90	90
β/°	96.6120(10)	90	90
γ/°	90	90	90
Volume/$Å^3$	2148.72(16)	3415.6(5)	3068.09(18)
Z	4	4	4
ρcalcg/cm^3	1.378	1.344	1.338
μ/mm^{-1}	1.178	0.774	0.85
F(000)	912	1456	1296
Crystal size/mm^3	0.06 × 0.06 × 0.03	0.258 × 0.186 × 0.174	0.302 × 0.189 × 0.167
Radiation	MoKα (λ = 0.71073)	MoKα (λ = 0.71073)	MoKα (λ = 0.71073)
2Θ range for data collection/°	4.792 to 54.99	5.466 to 55.042	5.608 to 57.456
Index ranges	-13 ≤ h ≤ 13, -14 ≤ k ≤ 14, -24 ≤ l ≤ 24	-38 ≤ h ≤ 38, -13 ≤ k ≤ 13, -14 ≤ l ≤ 14	-13 ≤ h ≤ 13, -14 ≤ k ≤ 15, -36 ≤ l ≤ 36
Reflections collected	51194	70220	45888
Independent reflections	4940 [R_{int} = 0.0559, R_{sigma} = 0.0309]	7856 [R_{int} = 0.1028, R_{sigma} = 0.0787]	7907 [R_{int} = 0.0281, R_{sigma} = 0.0227]
Data/restraints/parameters	4940/14/263	7856/18/413	7907/303/474
Goodness-of-fit on F^2	1.079	1.129	1.072
Final R indexes [I>=2σ (I)]	$R1^a$ = 0.0490, $wR2^b$ = 0.1324	$R1^a$ = 0.0912, $wR2^b$ = 0.1899	$R1^a$ = 0.0529, $wR2^b$ = 0.1382
Final R indexes [all data]	$R1^a$ = 0.0757, $wR2^b$ = 0.1536	$R1^a$ = 0.1328, $wR2^b$ = 0.2067	$R1^a$ = 0.0565, $wR2^b$ = 0.1410
Largest diff. peak/hole / e $Å^{-3}$	1.10/-0.69	1.05/-1.77	0.98/-0.88

$^aR_1=\Sigma||F_o|-|F_c||/\Sigma|F_o|$. $^bwR_2= |\Sigma w(|F_o|^2-|F_c|^2)|/\Sigma|w(Fo^2)^2|^{1/2}$

Table S1. *(Continued)*

**Compounds**	**4-β**, [Zn(tbia)(bphy)]$_n$ -β	**5-α**, {[Zn(pdia)(bphy)]·2DMF}$_n$ -α	**6-α**, {[Zn(dpdia)(bphy)]·2DMF}$_n$ -α
Identification code	2243685	2243706	2243687
Empirical formula	$C_{44}H_{44}N_8O_8Zn_2$	$C_{30}H_{32}N_8O_6Zn$	$C_{32}H_{36}N_8O_6Zn$
Formula weight	943.61	666.00	694.06
Temperature/K	100.00	100.00	100.00
Crystal system	monoclinic	orthorhombic	monoclinic
Space group	$P2_1$	$P2_12_12$	$P2_1/n$
a/Å	10.2525(5)	10.8129(2)	10.1801(5)
b/Å	11.0209(6)	30.0622(5)	11.4969(5)
c/Å	20.0963(10)	10.13660(10)	29.2282(15)
α/°	90	90	90
β/°	91.239(2)	90	93.760(2)
γ/°	90	90	90
Volume/Å^3	2270.2(2)	3295.00(9)	3413.5(3)
Z	2	4	4
ρcalcg/cm^3	1.38	1.343	1.351
μ/mm^{-1}	1.116	1.483	0.774
F(000)	976	1384	1448
Crystal size/mm^3	0.2 × 0.1 × 0.1	0.2 × 0.1 × 0.1	0.2 × 0.1 × 0.1
Radiation	MoKα (λ = 0.71073)	CuKα (λ = 1.54178)	MoKα (λ = 0.71073)
2Θ range for data collection/°	5.488 to 55.1	5.88 to 139.996	5.914 to 49.998
Index ranges	-13 ≤ h ≤ 13, -14 ≤ k ≤ 14, -26 ≤ l ≤ 26	-9 ≤ h ≤ 13, -36 ≤ k ≤ 36, -12 ≤ l ≤ 12	-12 ≤ h ≤ 12, -13 ≤ k ≤ 13, -34 ≤ l ≤ 34
Reflections collected	88853	25253	52956
Independent reflections	10450 [R_{int} = 0.0209, R_{sigma} = 0.0114]	6228 [R_{int} = 0.0501, R_{sigma} = 0.0414]	5837 [R_{int} = 0.0293, R_{sigma} = 0.0162]
Data/restraints/parameters	10450/43/566	6228/166/410	5837/17/478
Goodness-of-fit on F^2	1.105	1.096	1.118
Final R indexes [I>=2σ (I)]	R1^a = 0.0313, wR2^b = 0.0828	R1^a = 0.0799, wR2^b = 0.2337	R1^a = 0.0962, wR2^b = 0.2078
Final R indexes [all data]	R1^a = 0.0335, wR2^b = 0.0876	R1^a = 0.0858, wR2^b = 0.2408	R1^a = 0.0986, wR2^b = 0.2088
Largest diff. peak/hole / e Å^{-3}	1.40/-0.49	1.74/-0.77	1.62/-0.82

$^aR_1=\Sigma||F_o|-|F_c||/\Sigma|F_o|$. $^bwR_2= |\Sigma w(|F_o|^2-|F_c|^2)|/\Sigma|w(Fo^2)^2|^{1/2}$

Table S1. *(Continued)*

**Compounds**	**6-β**, $[Zn(dpdia)(bphy)]_n$ -β	**1**, (Refcode: FEFFAZ) $[Zn(nipa)(bphy)]_n$	**1**, activated $[Zn(nipa)(bphy)]_n$
Identification code	2243686	890037 (Published)	
Empirical formula	$C_{26}H_{21}N_6O_4Zn$		
Formula weight	546.86		
Temperature/K	136		
Crystal system	monoclinic		
Space group	$P2_1/n$	$P2_1/n$	$P2_1/n$
a/Å	10.1892(7)	10.1848(10)	10.2102(4)
b/Å	11.3316(9)	11.1036(11)	11.1494(5)
c/Å	24.901(2)	19.1820(18)	19.2753(9)
α/°	90	90	90
β/°	100.458(2)	94.397(2)	94.7880(10)
γ/°	90	90	90
Volume/Å^3	2827.3(4)	2162.87	2186.60(17)
Z	4		
ρcalcg/cm^3	1.285		
μ/mm^{-1}	0.908		
F(000)	1124		
Crystal size/mm^3	0.2 × 0.15 × 0.1		
Radiation	MoKα (λ = 0.71073)		
2Θ range for data collection/°	5.456 to 55.096		
Index ranges	-13 ≤ h ≤ 12, -14 ≤ k ≤ 14, -32 ≤ l ≤ 32		
Reflections collected	39082		
Independent reflections	6493 [R_{int} = 0.0658, R_{sigma} = 0.0549]		
Data/restraints/parameters	6493/0/336		
Goodness-of-fit on F^2	1.125		
Final R indexes [I>=2σ (I)]	$R1^a$ = 0.0988, $wR2^b$ = 0.2215		
Final R indexes [all data]	$R1^a$ = 0.1241, $wR2^b$ = 0.2327		
Largest diff. peak/hole / e Å^{-3}	0.70/-1.29		

$^aR_1=\Sigma||F_o|-|F_c||/\Sigma|F_o|$. $^bwR_2=|\Sigma w(|F_o|^2-|F_c|^2)|/\Sigma|w(Fo^2)^2|^{1/2}$

Table S2. Parameters of H-bonds within **sql** bilayers of as-synthesized phase in **1**-**6**.

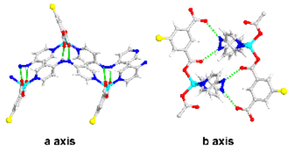	D...A lengths ($d_{N...O}$, Å)	D-H lengths (d_{N-H}, Å)	H...A lengths ($d_{H...O}$, Å)	D-H...A angles ($\angle_{N-H...O}$, °)
**1**	2.864(3), 2.857(3)	0.880(2), 0.879(2)	2.049(2), 1.992(2)	152.2(2), 171.2(2)
**2**	2.799(5), 2.832(5)	0.880(2), 0.880(2)	1.970(3), 1.978(3)	166.3(3), 154.9(3)
**3**	2.846(6), 2.845(5)	0.880(2), 0.880(2)	2.052(3), 1.968(3)	175.5(3), 150.2(3)
**4-α**	2.849(12), 2.838(12)	0.880(2), 0.880(2)	2.003(8), 2.012(8)	158.0(6), 158.4(6)
**5-α**	2.832(8), 2.821(8)	0.880(2), 0.880(2)	1.959(5), 1.963(5)	168.4(4), 166.0(4)
**6-α**	2.809(6), 2.840(7)	0.881(2), 0.879(1)	1.984(4), 2.019(4)	163.6(3), 148.8(3)

Table S3. Structural analysis of phases **1**-**6**.

	d_a (Zn…Zn) Å	d_b (Zn…Zn) Å	Torsion angle (C-N-N-C) °	Dihedral angle (∠ isophthalic plane and carboxyl plane) °		Dihedral angle (∠ isophthalic plane and dimethylphenyl plane) °
	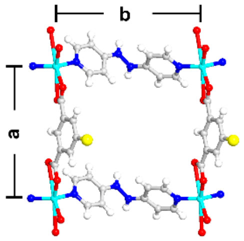		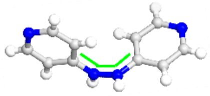	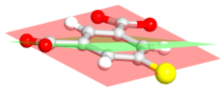		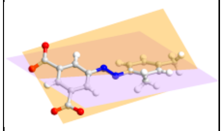
**1**	10.185(1)	11.104(1)	95.9(3)	9.0(2)	18.5(2)	
**2**	10.189(1)	11.199(1)	99.7(5)	10.6(3)	20.4(3)	--
**3**	10.211(7)	11.106(8)	96.3(5)	6.5(4)	16.6(3)	
**4-α**	10.152(2)	11.349(2)	101.9(10)	6.4(8)	10.2(1)	--
**4-α'**	10.203(1)	11.209(1)	98.3(5)	9.7(3)	21.2(3)	--
**4-β**	10.252(1)	11.021(1)	89.2(7)	11.4(5)	27.6(4)	--
**5-α**	10.137(1)	10.813(1)	88.8(8)	2.1(5)	15.5(6)	5.0(4)*
**6-α**	10.180(1)	11.497(1)	106.9(6)	8.4(4)	22.8(4)	5.2(3)
**6-β**	10.189(1)	11.332(1)	101.5(7)	18.1(4)	29.8(4)	31.7(4)

Dihedral angle was measured between the isophthalate aromatic ring plane and phenyl ring plane for* ***5-α**.*

Table S4. Non-covalent interactions in **1**, **2**, **3**, **4-β**, and **6-β**.

	π···π interactions $d_{Centroid\cdots Centroid}$ Å	C-H···π interactions $d_{C\cdots Centroid}$ Å				C-H···O interactions $d_{C\cdots O}$ Å			
	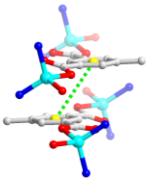	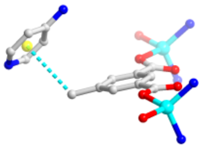				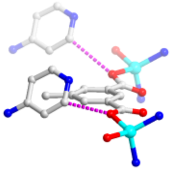			
**1**	4.137(1)	-	-	-	-	2.987(4)*	3.114(3)*	3.221(3)*	3.456(4)*
**2**	4.176(1)	3.543(6)*	-	-	-	3.115(5)*	3.450(5)*	-	-
**3**	3.811(1)	3.396(6)*	3.732(5)*	-	-	3.585(5)*	3.487(5)*	3.271(6)*	-
**4-β**	4.256(2)	3.673(7)	3.678(6)	3.800(5)	3.827(7)	3.675(7)	3.692(6)	3.787(5)	3.804(7)
**6-β**	3.825(1)	4.214(5)*	4.271(7)*	4.110(6)*	-	-	-	-	-

**Interactions appear in pairs with marked lengths.*

11. Reference

(1) Launay, J. P.; Tourrel-Pagis, M.; Lipskier, J. F.; Marvaud, V.; Joachim, C. Control of Intramolecular Electron Transfer by a Chemical Reaction. The 4,4'-Azopyridine/1,2-Bis(4-Pyridyl)Hydrazine System. *Inorg. Chem.* **1991**, *30* (5), 1033–1038.

(2) He, H.; Du, J.; Su, H.; Yuan, Y.; Song, Y.; Sun, F. Four New Metal–Organic Frameworks Based on Bi-, Tetra-, Penta-, and Hexa-Nuclear Clusters Derived from 5-(Phenyldiazenyl)Isophthalic Acid: Syntheses, Structures and Properties. *CrystEngComm* **2015**, *17* (5), 1201–1209.

(3) Dinker, M. K.; Zhao, K.; Dai, Z.; Ding, L.; Liu, X.-Q.; Sun, L.-B. Porous Liquids Responsive to Light. *Angew. Chem. Int. Ed.* **2022**, *61* (50), e202212326.

(4) Liu, X.M., Lin, R.B., Zhang, J.P. and Chen, X.M., Low-dimensional porous coordination polymers based on 1, 2-bis (4-pyridyl) hydrazine: from structure diversity to ultrahigh CO2/CH4 selectivity. *Inorg. Chem.* **2012**, *51*(10), 5686-5692.

(5) Sheldrick, G. M.; Bruker, A. X. S. Inc., Madison, WI, 2000;(b) GM Sheldrick. *Acta Crystallogr., Sect. A Fundam. Crystallogr* **2015**, *71*, 3–8.

(6) Krause, L.; Herbst-Irmer, R.; Sheldrick, G. M.; Stalke, D. Comparison of Silver and Molybdenum Microfocus X-Ray Sources for Single-Crystal Structure Determination. *J. Appl. Crystallogr.* **2015**, *48* (1), 3–10.

(7) Sheldrick, G. M. SHELXT–Integrated Space-Group and Crystal-Structure Determination. *Acta Crystallogr. Sect. A Found. Adv.* **2015**, *71* (1), 3–8.

(8) Dolomanov, O. V.; Bourhis, L. J.; Gildea, R. J.; Howard, J. A. K.; Puschmann, H. OLEX2: A Complete Structure Solution, Refinement and Analysis Program. *J. Appl. Crystallogr.* **2009**, *42* (2), 339–341.

(9) Sheldrick, G. M. Crystal Structure Refinement with SHELXL. *Acta Crystallogr. Sect. C Struct. Chem.* **2015**, *71* (Md), 3–8.

(10) Spek, A. L. PLATON SQUEEZE: A Tool for the Calculation of the Disordered Solvent Contribution to the Calculated Structure Factors. *Acta Crystallogr. Sect. C Struct. Chem.* **2015**, *71*, 9–18.

(11) Guide, C. Materials Studio 8.0. *BIOVIA, Dassault Systèmes* **2014**.

(12) Rappe, A. K.; Goddard III, W. A. Charge Equilibration for Molecular Dynamics Simulations. *J. Phys. Chem.* **1991**, *95* (8), 3358–3363.

(13) Metropolis, N.; Rosenbluth, A. W.; Rosenbluth, M. N.; Teller, A. H.; Teller, E. Equation of State Calculations by Fast Computing Machines. *J. Chem. Phys.* **1953**, *21* (6), 1087–1092.
